# Cross-modal associations between materic painting and classical Spanish music

**DOI:** 10.3389/fpsyg.2015.00424

**Published:** 2015-04-21

**Authors:** Liliana Albertazzi, Luisa Canal, Rocco Micciolo

**Affiliations:** ^1^Center for Mind/Brain Sciences (CIMeC), University of TrentoTrento, Italy; ^2^Department of Humanities, University of TrentoTrento, Italy; ^3^Department of Psychology and Cognitive Sciences, University of TrentoTrento, Italy

**Keywords:** connotative dimensions, cross-modal associations, music, painting, subjective judgments

## Abstract

The study analyses the existence of cross-modal associations in the general population between a series of paintings and a series of clips of classical (guitar) music. Because of the complexity of the stimuli, the study differs from previous analyses conducted on the association between visual and auditory stimuli, which predominantly analyzed single tones and colors by means of psychophysical methods and forced choice responses. More recently, the relation between music and shape has been analyzed in terms of music visualization, or relatively to the role played by emotion in the association, and free response paradigms have also been accepted. In our study, in order to investigate what attributes may be responsible for the phenomenon of the association between visual and auditory stimuli, the clip/painting association was tested in two experiments: the first used the semantic differential on a unidimensional rating scale of adjectives; the second employed a specific methodology based on subjective perceptual judgments in first person account. Because of the complexity of the stimuli, it was decided to have the maximum possible uniformity of style, composition and musical color. The results show that multisensory features expressed by adjectives such as “quick,” “agitated,” and “strong,” and their antonyms “slow,” “calm,” and “weak” characterized both the visual and auditory stimuli, and that they may have had a role in the associations. The results also suggest that the main perceptual features responsible for the clip/painting associations were hue, lightness, timbre, and musical tempo. Contrary to what was expected, the musical mode usually related to feelings of happiness (major mode), or to feelings of sadness (minor mode), and spatial orientation (vertical and horizontal) did not play a significant role in the association. The consistency of the associations was shown when evaluated on the whole sample, and after considering the different backgrounds and expertise of the subjects. No substantial difference was found between expert and non-expert subjects. The methods used in the experiment (semantic differential and subjective judgements in first person account) corroborated the interpretation of the results as associations due to patterns of qualitative similarity present in stimuli of different sensory modalities and experienced as such by the subjects. The main result of the study consists in showing the existence of cross-modal associations between highly complex stimuli; furthermore, the second experiment employed a specific methodology based on subjective perceptual judgments.

## Introduction

In recent years the field of perception studies has seen an increasing amount of research showing the tendency for a sensory feature, or attribute, in one modality to be matched with a sensory feature in another modality (Simner et al., 2005, 2011; Sagiv and Ward, 2006; Ward et al., 2006a,b; Cohen Kadosh et al., 2009. For a review see Spence, 2011). The phenomenon had already been pointed out by Köhler, who showed the tendency of the general population systematically to associate visual and auditory attributes (the so-called “takete-maluma” phenomenon) (Köhler, 1929; Gallace et al., 2011; Nielsen and Rendall, 2011). Initially prompted by interest in the field of synesthesia (Wicker, 1968; Melara and O'Brien, 1987; Cytowic, 1995; Baron-Cohen and Harrison, 1997; Ward and Simner, 2003; Simner et al., 2006), studies then considered similar phenomena occurring in the general population (Martino and Marks, 2001; Maurer and Mondloch, 2005; Sagiv and Ward, 2006; Spector and Maurer, 2008, 2011; Parise and Spence, 2009; Deroy et al., 2013; Deroy and Spence, 2013). In regard to the nature of such associations, Spence has distinguished among structural correspondences (due to neural correlates, hence potentially universal), statistical correspondences (due to learning, hence potentially influenced by different environments), and semantic correspondences (due to language influence, hence potentially different among cultures) (Spence, 2011). Recently, a growing number of researchers have sought to explain synesthetic and cross-modal associations in semantic more than sensory terms by re-evaluating the possible role of cognitive factors in the associations. In particular, the question has been raised in regard to inducers that take the form of concepts—such as the days of the week or the months—usually associated with colors. In short, since inducers have a conceptual nature, it has been asked whether a full account of synesthesia should not go beyond the standard sensory-sensory approach (Dixon et al., 2006; Simner and Ward, 2006; Ward et al., 2007; Eagleman, 2012; Jürgens and Nikolić, 2012, 2014; Mroczko-Wąsowicz and Werning, 2012; Simner, 2012; Ward, 2013; Mroczko-Wąsowicz and Nikolić, 2014).

### Bottom up and top down explanations

The opposition between the sensory interpretation (bottom-up, i.e., sense-driven) and the conceptual interpretation (top-down, i.e., concept driven) of synesthetic associations has arisen within the classical framework of cognitive science, which counterposes the two levels of information processing. Consequently, the former interpretation has sought to explain the associations in terms of direct synaptic connections between neurons (representing the inducer and the concurrent); the latter is based on high-level processes due to language, culture, abstract symbolization, learning, etc. (Ward et al., 2007). The second interpretation, however, would seem better suited to explaining cases of color sequence synesthesia (Simner et al., 2006; Tomson et al., 2013) and spatial sequence synesthesia (Sagiv et al., 2006; Eagleman, 2010), where the names of time units and ordinal categories are involved. This second interpretation, which considers cases of synesthesia occurring independently of external inducers, has taken the name of ideasthesia (Meier, 2013; Jürgens and Nikolić, 2014 for a thorough discussion of the topic see Mroczko-Wąsowicz and Nikolić, 2014).

There is also another interpretation. It rests on a not necessarily linguistic or symbolic conception of semantics. This third approach, of Gestalt derivation (Albertazzi, 2013), explains associations in terms of *patterns of qualitative similarity* present in different sensory modalities and perceived as such: for example, hot and cold, sad and happy, and pleasant and unpleasant, are connotative properties of both sounds and colors. This therefore concerns, not semantic information projected top-down into other domains, but qualities intrinsic to perceived phenomena. This position obviously does not preclude investigation of *correlations* at neuronal level or of the presence of cognitive dimensions due to learning, language, symbolization, etc. This interpretation has been adopted in studies on the associations between color and shape in the general population (Dadam et al., 2012; Albertazzi et al., 2013, 2014, 2015).

Whatever viewpoint is adopted in interpreting the phenomenon, there is growing interest in cross-modal associations both within synesthesia (Simner, 2012; Ward, 2013) and in the general population (Deroy and Spence, 2013).

In the field of cross-modal associations occurring in the general population, a perceptual attribute which proves to play an important role is color. In fact, it has been shown that color is associated with olfaction (Gilbert et al., 1996; Kemp and Gilbert, 1997; Demattè et al., 2006; Hanson-Vaux et al., 2013; Levitan et al., 2014), touch (Ludwig and Simner, 2013), and acoustics (Ward et al., 2006a; Moos et al., 2013, 2014).

### Cross-modal associations between visual and auditory stimuli

As regards studies on associations between visual and auditory stimuli, initially considered were predominantly single tones by means of psychophysical methods and forced choice responses (Walker, 1987). More recently, the relation between visual and more complex auditory stimuli (i.e., music clips) has also been tested (Tan and Kelly, 2004; Küssner, 2013b), and free response paradigms have also been accepted (Reybrouck et al., 2009). In particular, the task in Tan and Kelly (2004) was to create marks or drawings that visually represented five short orchestral compositions, and to write essays explaining their graphic representations; while in Küssner (2013b) the task was to visualize sound and music by creating representations with an electronic graphics tablet (in two different experimental conditions, i.e., drawing during and after the sounds). In their experiment, Tan and Kelly tested musically trained and untrained subjects, and found a difference between them: i.e., the musically trained participants provided abstract representations, such as lines of symbols, while the untrained participants produced pictorial representations, such as images or pictures telling a story. A second difference consisted in the fact that trained participants focused more on musical characteristics (such as theme, mode, changes in pitch, etc.), while untrained participants focused more on emotions.

It has also been found that music in the major mode more closely matches lighter colors than does music in the minor mode (Bresin, 2005), while faster music in the major mode more closely matches more saturated and lighter colors than does slower music in the minor mode (Palmer et al., 2013). In particular, Bresin's study explicitly addressed the role of expressivity in music, which was verified by testing the association made by expert subjects between colors and performances of classical music. The results of this study showed that participants used different color profiles to classify the same piece of music, but these differences depended mainly on the performance and on the instrument. The study by Palmer et al. (2013) instead tested participants of different cultures (United States and Mexico), and found that in both cultures, faster music in the major mode produced color choices that were more saturated, lighter, and yellower, whereas slower, minor music produced the opposite pattern. Similarly, other studies have been conducted in order to explain the association between visual and auditory stimuli on the basis of their shared emotional content, such as the association between happy music and happy colors (Whiteford et al., 2013; Langlois et al., 2014).

As is well known, cross-modal and cross-dimensional associations have always played a role in aesthetics. Recently, the association between color and shape has been experimentally tested by studies relating to Kandinsky's hypothesis of a systematic association between geometrical shapes and colors (Droste, 1990; Lupton and Miller, 1991; Jacobsen, 2002; Kharkhurin, 2012; Albertazzi et al., 2013, 2015; Makin and Wuerger, 2013; Chen et al., 2015). Besides the above-mentioned shape/color association in Kandinsky, the analogy between scales of colors and scales of notes is a major component of the harmony theories developed by Klee (1956), Kandinsky (1926/1994), and Itten (Itten et al., 1970; Gage, 1999) with particular regard to Schönberg (Schönberg and Kandinsky, 1980; Bidaine, 2004). In our study, and following previous studies of ours dealing with aesthetics (Albertazzi et al., 2013, 2015), we tested the association in the general population between some artistic works in painting and some pieces of classical music, in order to evaluate whether systematic cross-modal associations occur among stimuli of high complexity. Specifically, we tested whether images with varying perceptual characteristics and contents led to consistent associations with the music clips, and what attributes might be responsible for the phenomenon. The choice of the images and of the clips was based on the hypothesis that their artistic modes of expressions (the coloratura of the flamenco and the materic style of the paintings), and a series of connotative properties holding for colors and tones (like weak and strong, calm and agitated) play a role in the associations. Precisely, the specific coloratura (Tonkolorit) of flamenco music is characterized by very brief sharp notes and a minor scale. As to materic (or material) painting, this is painting realized with a great quantity of pictorial material, and characterized by a thick and tendentially 3D pictorial surface. The study that may show an affinity with ours was conducted in the 1930s by Cowles (1935), who also made use of complex stimuli (8 pieces of classical music, although composed by different musicians, and 8 paintings by various well-known artists), and with expert and non-expert participants. There are differences with our study, however, both in the number and in the kind of stimuli: in Cowles (1935) the pictures were mainly landscapes or scenes with simple content, without uniformity of style; the auditory stimuli were taken from works by different composers, differing in character, although there were no more than slight variations in volume, tempo, or tone quality. Finally, the aim of one of the two experiments conducted in Cowles' study, differently from ours, was to verify whether similar affective moods were found between the musical selections and the pictures.

## The study

The purpose of our research was to test whether the general population exhibits cross-modal associations between complex stimuli of two different modalities (vision and sound). Specifically, the aim of our research was to test whether significant associations existed between a series of paintings and a series of clips of classical (guitar) music, and whether these associations were consistent when evaluated on the same subjects. The research also sought to evaluate whether the findings were confirmed on different subjects with different backgrounds and expertise. Our expectation was that, if found, these associations would be consistent from one subject to another, suggesting a predisposition to perceive specific cross-modal natural associations between complex visual and auditory stimuli.

The selection of the paintings and the music clips was discussed with the painter (Matteo Boato: http://www.matteoboato.net/), who is also a musician and who provided a description of the individual art works and the characteristics of the music clips selected. The choice of Boato's works was made (apart from personal preference) on the basis of their characteristics of high chromaticity and saturation. Our hypothesis was that corresponding to these visual characteristics are similar patterns in the acoustic modality as to vibrato, coloratura, and quick tempo: for example, we expected that a quick tempo would correspond to a very chromatic and saturated red or yellow. Specifically, the hypothesis was that the association, if found, would be due to multisensorial and connotative features present in both the visual stimuli and the auditory stimuli, such as warmth/coldness, brightness/darkness, sadness/happiness, softness/hardness, etc. The prediction was therefore that the subjects would make systematic associations between the paintings and the music, and that the associations would be due to the presence of similar features in the paintings and the music, as also evidenced by the semantic differential. Because of the complexity of the stimuli, we tried to keep the maximum amount of uniformity possible. The purpose of using works by the same painter as stimuli was to maintain the same style (materic painting) and composition (expressionist) notwithstanding the diversity of content and colors (achromatic and mainly chromatic paintings depicting landscapes and figures were tested). The purpose of using clips from the same repertoire was to maintain the coloratura of classical Spanish flamenco music. The clips were instead chosen for their specific musical features, such as having a strong, hard, agitated sound and a quick tempo (*presto*) (for example, *Asturias* by Albéniz). The recorded music clips were performed by Boato himself. Finally, we did not test individual preferences because it was not an objective of our experiment.

## Methods

### Participants

Sixty-three participants volunteered for two experiments: 38 women and 25 men (mean age: 22.6 years; standard deviation: 3.5; median: 22 years). All participants were recruited by e-mail from students in the Department of Cognition and Education Sciences, University of Trento, Italy. The address list of the students was provided by the student office. We firstly sent a mail asking the students to adhere to the experiment, mentioning that we were looking for people with a background in music, people with a background in art, and non-expert people. We didn't ask for professional people, however. When we contacted the students who adhered to the experiment, we decided to accept people who had a public or private artistic education in music for at least 4 years, people who had a private or public education in art, and non-expert people. The questionnaire reported this information. The subjects were also asked about a possible conscious synesthesia (Palmer and Schloss, 2010; Albertazzi et al., 2013, 2015; Palmer et al., 2013). The only exclusion criterion was self-reported defective color vision or acoustic impairment. After the experiment, the subjects were asked whether they had previously known the paintings and the pieces of music that they evaluated. For all the subjects the stimuli were totally new.

The first experiment was performed using the semantic differential on a unipolar rating scale of adjectives. It was decided to use a unipolar scale instead of the classic bipolar one of the Osgood semantic differential (Osgood, 1956) because the bipolar scale is not always one-dimensional. Sixty-one subjects participated in the experiment (two subjects who did not complete the experiment were excluded from the analysis). The second experiment evaluated the association between visual and auditory stimuli and was completed by all the 63 subjects. We tested non-experts (31), music experts (20), and art experts (12), the purpose being to investigate a possible influence of expertise on the associations. Individuals with training in private or public schools were considered expert participants in the present study. All the subjects signed an informed consent form. The experiments reported here complied with the ethical guidelines of the University of Trento.

### Procedure

The experiment was performed in a laboratory with constant and controlled lighting conditions (230–250 lux) in the room, correlated color temperature 3400K, halogen lamp). The visual stimuli appeared on a Quato Display 242ex (Intelli Prof 242 excellence) 24″ screen (51.8 × 32.4 cm visible area); the auditory stimuli were administered through Sennheiser HD580 Precision headphones. Automatic 48 bit USB-hardware calibration with 3 × 16 bit 3D Look-Up Table and luminance inside the monitor, dedicated luminance stability circuit, UDACT display analysis built-in; the measurement device was a 4-channel Silver Haze Pro colorimeter. The resolution used was 1920 × 1200 pixels (the native and the maximum possible for the monitor Display Quato 242.

Participants were seated at a desk. The distance from the center of the screen to the eye was about 65 cm. Chin supports were not used, but during each session the postures of the participants were checked and corrected if their chests approached the screen or their backs were hunched.

### General materials

The materials consisted of a series of 15 paintings (by the same painter), a series of 15 music clips and a list of 22 adjectives. The titles of the paintings were: (1) “Padova, 2007,” (2) “Verona, 2009,” (3) “Mantova, 2009,” (4) “Trento, 2008,” (5) “Full Moon,” (6) “The Circle,” (7) “Trento, 2006,” (8) “Burano, 2009,” (9) “Sky of Fields,” (10) “Sea II,” (11) “Land—Hora et Labora,” (12) “In Dream II,” (13) “In Dream 2006,” (14) “Leopard,” (15) “Matilada and Beatrice” (see Supplementary Material, reproduction permitted by Boato). For presentation of the digital images a high-resolution digital transcription was performed by an expert in the graphic reproduction of works of art.

The clips (performed by the same player) were taken from the following musical works: (1) Heitor Villa Lobos, *Prelude n. 4;* (2) Heitor Villa Lobos, *Mazurka, Suite populaire brésilienne;* (3) Francisco Tárrega, *Recuerdos de la Alhambra;* (4) Isaac Albéniz, *Asturias—Part I*; (5) Fernando Sor, *Variations on a theme by Mozart—II var;* (6) Gaspar Sanz, *Canarios, Suite Española*; (7) Fernando Sor, *Variations on a theme by Mozart—Theme;* (8) Fernando Sor, *Variations on a theme by Mozart—I var;* (9) Manuel Ponce, *Giga;* (10) Gaspar Sanz, *Espanoletas, Suite Española*; (11) Heitor Villa Lobos, *Prelude n. 5—Part I;* (12) Heitor Villa Lobos, *Prelude n. 5—Part II;* (13) Isaac Albéniz, *Leyenda, Asturias*; (14) Heitor Villa Lobos, *Study n. 6;* (15) Francisco Tárrega, *Arabian caprice*.

**Figure 1:**
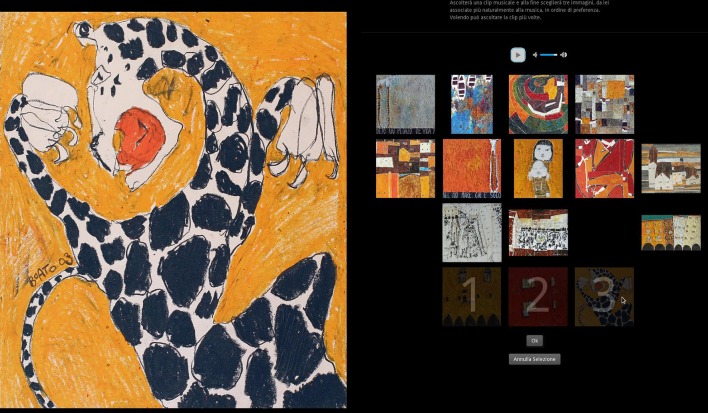
**Example of a painting selected in association with a given music clip (The arrow points where to click to hear the music clip again)**.

The assessments of the adjectives were arranged on a continuous scale between 0 and 1024. We selected for the experiment mainly adjectives that could be applied to both music and paintings. The experiment was preceded by a pilot test with the same characteristics as the experiment itself but a much longer list of adjectives. The original list of adjectives included 49 items evaluated by 35 subjects. After a correlational study, the list of adjectives was shortened to include 22 items. The final list of adjectives (presented in Italian) was the following: slow, quick, agitated, calm, happy, sad, warm, cold, heavy, light, continuous, rhythmic, strong, weak, dark, bright, hard, soft, impression of horizontality, impression of verticality, adagio, presto (the two last items were left in the adverbial form as they are in Italian). As to the chromatic dimensions, neither hue nor saturation were considered (all the paintings were uniformly drawn with very saturated hues), but rather the dimensions of warmth (warm/cold) and brightness (light/dark) (relying on the contrast between the fragments of colors and the painted background used by the painter). The choice of dimensions was due to their perceptual salience and to the fact that they are the most meaningful dimensions in cross-modal associations where color is involved. The asymmetric choice of having the subjects listen to a music clip and asking them to associate three paintings with them, and not vice versa, was dictated by the complexity of the task, which was of considerable duration (about an hour and a half, with a pause). We also hypothesized that asking the subjects to look at the paintings and associate three music clips from the classical guitar repertoire with them would have been an excessively burdensome task. In fact, it would have required listening to 15 clips sequentially (although in random order) for each painting. Instead, as shown in Figure 1, the 15 paintings were seen all together.

### Experiment 1

The experiment was performed using the semantic differential on a unidimensional rating scale of adjectives. First the individual images (in random order) were presented on the screen and then each music clip was executed (also in this case, the order of presentation was randomized). Participants were told that they would first see a set of images (each was displayed on the screen for 10 s) and then hear a series of music clips (each lasting 60 s). For each stimulus the subject had to evaluate, on a continuous scale, his/her degree of agreement with a series of adjectives. Participants were given the following written instructions for the task:

*You will be presented with images on the screen or music clips through your headphones accompanied by a series of adjectives in succession. You should evaluate these adjectives with reference to the image or music presented. Evaluation of the adjective will be made on a continuous scale. You should prefer accuracy to promptness of response*.

**Figure 2:**
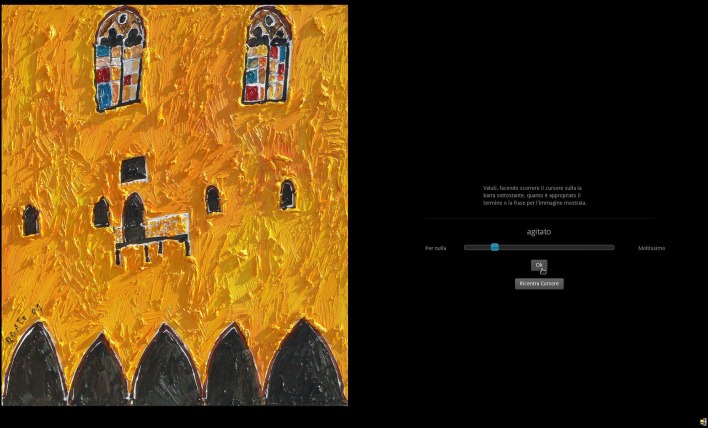
**Example of a painting to be evaluated according to a series of adjectives**.

The purpose of the experiment was to check whether complex images and music clips with varying perceptual characteristics led to consistent choices of adjectives. Images were shown one by one (in random order) on the left half of the screen, while on the right half of the screen participants saw one after the other the adjectives presented randomly (Figure 2). The same occurred with the music clips, which could be heard by clicking on a button positioned on the left side of the screen.

### Experiment 2

The purpose of the second experiment was to check whether images with varying perceptual characteristics and contents led to consistent associations with music clips taken from the repertoire of classical (guitar) music. Each subject saw a series of images of paintings in preview on the screen. The subject clicked on a specific image, which thus appeared in full screen mode, and likewise with the other images, in no particular order. The subject viewed the images while simultaneously listening to a music clip. The subject had to choose the image(s) that s/he most naturally associated with that music. S/he could list up to three images associated with the clip, arranging them in order of appropriateness from 1 to 3 in three different boxes at the bottom on the screen (Figure 1). The subject could go back to re-view images already seen, and s/he could also listen repeatedly to the music clip. Once the association had been decided, the images selected were transported down into one of three boxes, depending on the degree of association, in order from 1 to 3. Once the choice had been confirmed, it could not be changed, and the task continued with re-presentations of all the images and further music clips until the latter were exhausted.

Participants were given the following written instructions for the task:

*You will see a series of images of paintings in preview on the screen. Click on one of them, which will appear in full screen mode, and then do likewise with the other images. At the same time, you will hear a music clip. Select which image(s) you most naturally associate with the music. You can go back to re-view images already seen, and also to hear the music clip again. You can list up to three images associated with the music, placing them in order of appropriateness from 1 to 3. Once you have confirmed your choice, it cannot be changed, and the task will continue with further music clips until there are none left. You should prefer accuracy to promptness of response*.

### Statistical methods

Associations between quantitative variables were evaluated by means of the non-parametric “rho” correlation coefficient. The chi-square test for a contingency table was employed to evaluate the associations between the paintings and the music clips. A residual analysis was performed to identify which painting/clip combinations were significant (Canal and Micciolo, 2013). Analyses were performed with R 3.0.0 software (R Core Team, 2013).

### Results

#### Experiment 1

Table 1 reports the mean rating values for each word-painting pair given by the 61 participants. Means range between 186 and 842. This latter value was obtained when considering painting number 3 (“Mantova, 2009”) and the adjective “bright”; therefore this painting was considered the most luminous. The minimum value was obtained when considering painting number 14 (“Leopard”) and the adjective “weak”; therefore this painting was considered the least weak of the 15 paintings.

**Table 1:** **Mean ratings for each word and for each painting given by the 61 participants**.

**Painting^◦^**															
Word	1	2	3	4	5	6	7	8	9	10	11	12	13	14	15
Adagio	376	427	511	457	557	377	502	401	469	430	393	493	427	238	520
Presto	582	548	393	435	270	476	370	445	415	492	509	436	401	585	325
Agitated	619	617	362	533	236	613	347	487	457	628	608	460	447	777	325
Calm	362	426	599	433	701	401	629	471	552	469	319	500	512	188	609
Bright	527	696	842	767	507	691	720	649	677	704	738	582	732	724	748
Dark	444	348	254	284	506	268	275	368	272	254	271	273	341	332	373
Cold	628	393	258	239	590	196	358	339	463	439	244	593	235	291	291
Warm	254	509	762	709	373	794	609	575	494	547	724	373	749	624	653
Continuous	519	583	546	470	578	458	595	643	530	479	710	530	577	453	534
Rhythmic	563	591	401	447	449	584	555	621	686	522	721	448	435	527	395
Happy	363	499	570	477	252	535	529	413	447	553	664	424	446	395	502
Sad	556	361	320	324	652	282	391	469	376	339	268	419	449	295	434
Hard	480	424	445	565	480	494	477	550	502	384	427	407	444	590	367
Soft	303	401	441	374	330	373	387	311	327	429	423	466	409	289	487
Heavy	397	425	448	576	549	437	482	547	417	342	470	342	517	467	482
Light	447	411	439	350	407	425	462	309	480	532	356	529	411	330	437
Horizontality	329	617	455	401	696	467	514	423	497	361	429	248	270	272	310
Verticality	718	549	638	571	587	679	703	761	661	694	525	800	810	652	718
Quick	577	542	335	440	243	553	367	427	396	544	630	438	405	689	266
Slow	350	398	511	412	674	370	481	465	505	465	341	496	499	218	576
Strong	482	507	566	686	369	688	506	556	519	563	629	444	631	733	469
Weak	323	316	307	251	494	258	363	321	377	290	268	374	266	186	391

◦ *See text for the correspondence between the ID number and the title of the painting*.

Table 2 reports the mean rating values for each word-clip pair given by the 61 participants. Means range between 133 and 864. This latter value was obtained when considering clip no. 14 (Villa Lobos, *Study n. 6*) and the adjective “agitated”; therefore this clip was considered the most agitated. The minimum value was obtained when considering clip no. 8 (Fernando Sor, *Variations on a theme by Mozart—I var*) and the adjective “dark”; therefore this clip was considered the least dark of the 15 clips.

**Table 2:** **Mean ratings for each word and for each clip given by the 61 participants**.

**Music Clip^◦^**																
Word	1	2	3	4	5	6	7	8	9	10	11	12	13	14	15
Adagio	444	312	595	182	333	206	350	221	269	603	409	597	274	163	392
Presto	489	620	392	655	580	682	583	696	664	410	559	349	592	696	549
Agitated	598	661	346	805	645	685	500	634	725	370	500	397	646	864	578
Calm	453	366	600	137	327	209	415	238	254	597	423	570	308	162	406
Bright	423	646	376	376	631	818	790	793	592	505	659	358	545	459	499
Dark	538	336	512	535	272	137	156	133	366	392	225	533	357	506	438
Cold	443	383	468	480	335	215	226	279	361	344	332	412	385	456	419
Warm	492	558	458	460	582	713	661	657	531	546	579	529	516	467	488
Continuous	448	588	736	623	624	633	691	667	621	614	558	588	489	607	521
Rhythmic	543	651	491	778	669	790	646	785	742	518	634	508	638	767	581
Happy	320	570	269	411	613	780	781	820	553	476	657	306	516	456	429
Sad	636	408	682	485	410	147	167	188	397	532	261	678	437	430	551
Hard	414	382	355	617	366	322	213	273	459	296	302	398	475	632	410
Soft	390	459	526	233	501	443	584	477	393	595	505	541	343	248	431
Heavy	446	333	352	474	280	250	183	180	359	313	243	441	336	522	317
Light	416	525	512	354	543	585	742	641	496	617	563	497	444	312	504
Horizontality	494	535	669	430	524	460	655	483	584	574	574	636	461	385	518
Verticality	585	587	382	651	485	609	414	578	479	434	492	380	536	649	562
Quick	604	745	377	817	714	806	687	819	758	401	656	363	706	830	636
Slow	462	263	598	157	241	166	254	159	214	556	295	647	286	137	348
Strong	537	478	374	727	485	624	406	472	582	361	418	422	554	730	481
Weak	305	336	472	160	303	195	342	236	262	497	334	447	274	217	366

◦ *See text for the correspondence between the ID number and the title of the clip*.

To evaluate the degree of association between the semantic rating (i.e., considering the mean ratings of the 22 words) of one selected painting and one selected clip, non-parametric rho correlation coefficients were calculated. The results are shown in Table 3 (the rows contain the 15 music clips, the columns the 15 paintings).

**Table 3:**
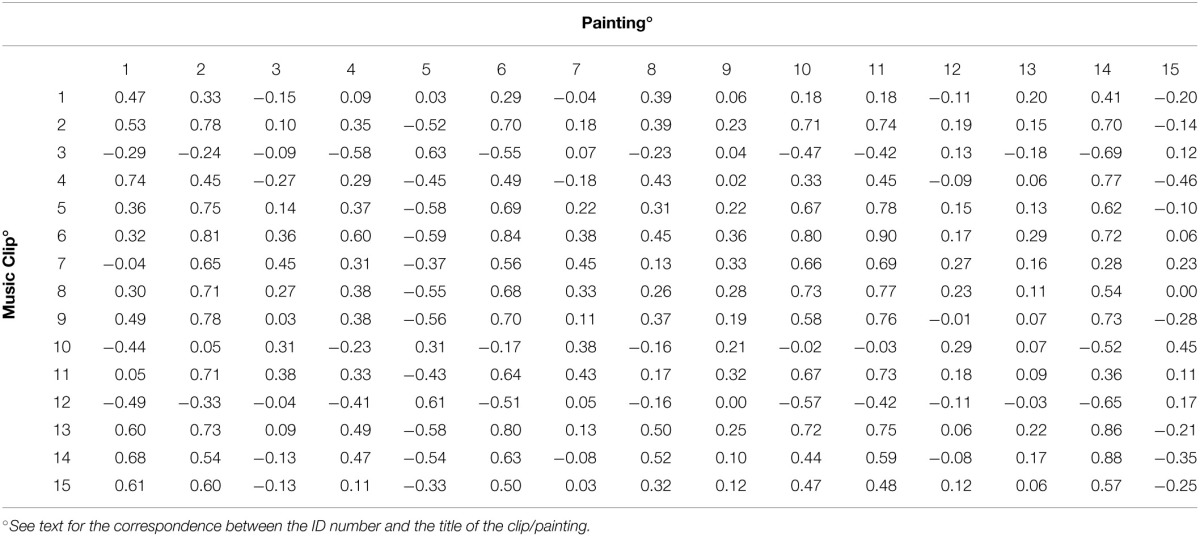
**Correlation coefficients (Spearman's rho) for each combination clip/painting evaluated employing the semantic ratings reported in Tables 1, 2**.

*^◦^See text for the correspondence between the ID number and the title of the clip/painting*.

These correlations ranged between -0.69 and 0.90. This latter value was obtained when considering the mean ratings of the 22 words given to painting no. 11 (“Land—Hora et Labora”) (see Table 1) and to clip no. 6 (Gaspar Sanz, *Canarios*) (see Table 2). The highest negative correlation was found between painting no. 14 (“Leopard”) and clip no. 3 (Francisco Tárrega, *Recuerdos de la Alhambra*).

#### Experiment 2

Table 4 shows the results of Experiment 2.

**Table 4:**
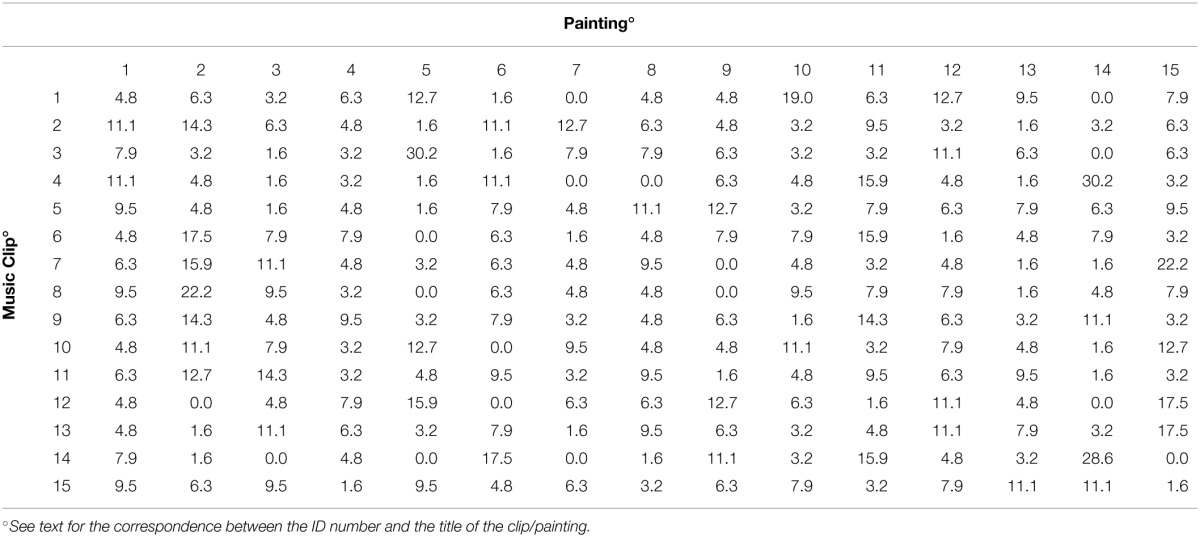
**For each clip listened to, the number of times (in percentage) each painting was chosen is reported (Each row of the table sums up to 100)**.

*^◦^See text for the correspondence between the ID number and the title of the clip/painting*.

For each clip listened to (shown in the rows of the table), the percentage of painting choices is reported (considering only the first choice of a painting). It seems evident from visual inspection of the table that some paintings were more frequently associated with a given clip: for example, 30.2% of participants associated painting no. 5 (“Full Moon”) with clip no. 3 (Francisco Tárrega, *Recuerdos de la Alhambra*). On the other hand, some paintings were less frequently associated with a given clip; for example, none of the participants associated painting no. 14 (“Leopard”) with clip no. 1 (Villa Lobos, *Prelude n. 4*). The chi-square test revealed that the association between the variables “painting” and “clip” cannot be considered random but instead systematic (chi-square = 517; d.f. = 196; *p* < 0.001). Given that the lowest expected frequency was less than 5, a Monte Carlo simulation was performed which confirmed the significance of the association (*p* < 0.001).

Since the test did not indicate which clip was associated (positively or negatively) with which painting, a residual analysis was performed. A standardized form of the residual was employed. This behaves like a normal deviate to determine whether the residual is large enough to indicate a departure from a random choice. In this case, there is only about a 5% chance that any particular standardized residual exceeds 1.96 in absolute value. When we inspected 225 cells, about 11 residuals (i.e., 5% of 225) could have been so large solely because of random variation. On the other hand, as can be seen in Table 5, there were 40 residuals greater than 1.96 in absolute value.

**Table 5:**
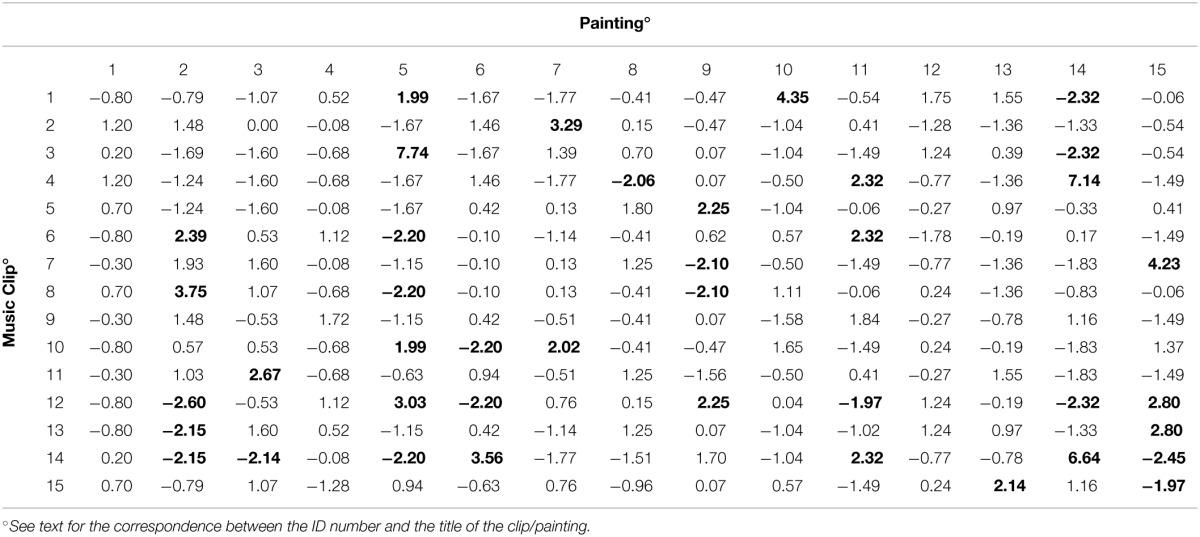
**Standardized residuals of the contingency table between the music clips and the first choice of a painting (Residuals greater than 1.96 in absolute value are shown in bold)**.

*^◦^See text for the correspondence between the ID number and the title of the clip/painting*.

Overall, there were 22 residuals greater than 1.96, and 18 residuals lower than -1.96. A positive residual means that the selected clip “attracted” the corresponding painting; a negative residual means that the selected clip “repelled” the corresponding painting.

There were five clips which showed a very strong attraction (a residual greater than 4). Clip no. 3 (Tárrega, *Recuerdos de la Alhambra*) was strongly associated with image no. 5 (“Full Moon”); clip no. 4 (Albéniz, *Asturias*) was strongly associated with image no. 14 (“Leopard”); clip no. 14 (Villa Lobos, *Study n. 6*) was strongly associated with image no. 14 (“Leopard”); clip no. 1 (Villa Lobos, *Prelude n. 4*) was strongly associated with image no. 10 (“Sea II”); clip no. 7 (Sor, *Variations on a theme by Mozart*) was strongly associated with image no. 15 (“Matilada and Beatrice”).

On the other hand, the negative associations were weaker; the lowest residual was -2.60. Clip no. 12 (Villa Lobos, *Prelude n. 5—Part II*) was negatively associated with image no. 2 (“Verona, 2009”); clip no. 14 (Villa Lobos, *Study n. 6*) was negatively associated with image no. 15 (“Matilada and Beatrice”); clips no. 1 (Villa Lobos, *Prelude n. 4*), no. 3 (Tárrega, *Recuerdos de la Alhambra*), and no. 12 (Villa Lobos, *Prelude n. 5—Part II*) were all negatively associated with image no. 14 (“Leopard”).

#### A comparison between the results of experiment 1 and experiment 2

To evaluate if and to what extent the “direct” associations found in Experiment 2 were in agreement with the correlations in terms of semantic differential (Experiment 1), we counted how many times the sign of the “significant” residuals (i.e., residuals greater than 1.96 in absolute value) shown in Table 5 for the 40 clip/painting combinations was the same as the corresponding correlation shown in Table 3. For 21 combinations, both the residuals and the correlations were positive, showing that, when a particular painting was attracted by a given clip, the 22 words had similar ratings. On the other hand, for 12 combinations both the residuals and the correlations were negative, showing that, when a particular painting was repelled by a given clip, the 22 words had opposite ratings. In the remaining seven combinations, the sign of the residual and the sign of the correlation disagreed. If the clip/painting associations shown in Table 5 randomly agreed with the correlations shown in Table 3, a total of 20 combinations would have the same sign and 20 combinations would have different signs. An exact binomial test yielded a significant result (*p* < 0.001), in contrast with the hypothesis that the associations found were essentially random.

Furthermore, the correlation between the values reported in Table 3 and all the standardized residuals shown in Table 5 was significantly different from zero (rho = 0.338; *p* < 0.001). Therefore, at least in part, the painting/clip association could be explained by similar perceptual characteristics.

Quite similar results were found when the analyses described above were performed considering all the three paintings selected for a given clip. The final correlation coefficient was 0.365 (0.338 was found when only the first painting chosen was considered).

The subjects who participated in the experiment were classified into three groups: music experts, painting experts, and non-experts. When all the analyses were repeated selecting only the subjects of the same group, similar results were found. When music experts were selected, the correlations were 0.293 (considering only the first painting chosen) and 0.341 (all the three paintings chosen). When painting experts were selected, the correlations were, respectively, 0.210 and 0.319. Painting/clip association in terms of similar perceptual characteristics was confirmed also within the three groups.

## Discussion

The study tested whether the general population exhibits cross-modal associations between complex stimuli of two different modalities, and specifically between a series of paintings and a series of clips of classical (guitar) music. The test was conducted with subjects who were both expert and non-expert in visual and musical arts.

The study tested the association in two experiments. One was conducted using the semantic differential on a unidimensional rating scale of adjectives; the other was based on subjective judgments on the association between visual and auditory stimuli. The hypothesis was that the association, if found, would be linked to the presence of characteristics of the paintings and the music clips, perceived as such by the subjects, and evidenced also when evaluated by means of the semantic differential. Due to the experimental nature of the study, the link between the two experiments cannot be consistently found for each clip/image couple; in some cases such a link may not be consistent. Overall, the results show the existence of an association between paintings and music clips among experts in music, experts in painting, and subjects with no artistic training, within each group and overall.

**Figure 3:**
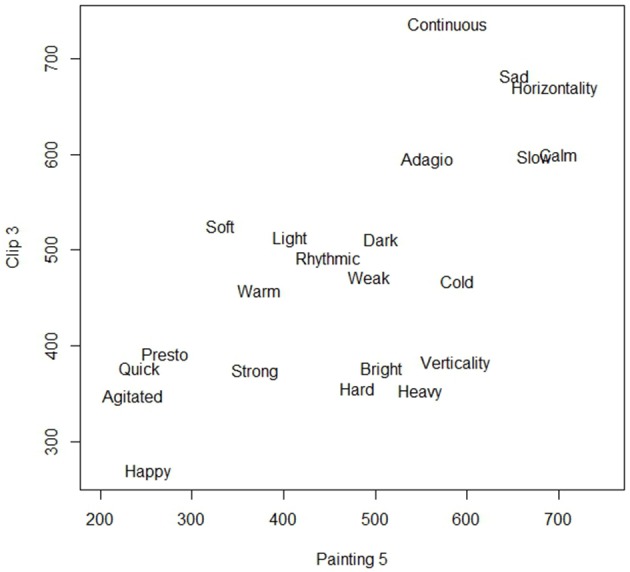
**Semantic rating of clip 3 (Francisco Tárrega, *Recuerdos de la Alhambra*) and painting 5 (“Full Moon”)**.

These results were consistent when considering both the first painting chosen and all the three paintings selected for a given clip. Specifically, there were five clip/image couples for which a very strong attraction was found: specifically, clip no. 3 (Francisco Tárrega, *Recuerdos de la Alhambra*) was strongly associated with image no. 5 (“Full moon”); clip no. 4 (Isaac Albéniz, *Leyenda*) was strongly associated with image no. 14 (“Leopard”); clip no. 14 (Villa Lobos, *Study n. 6*) was strongly associated with image no. 14 (“Leopard”); clip no. 1 (Villa Lobos, *Prelude n. 4*) was strongly associated with image no. 10 (“Sea II”); clip no. 7 (Fernando Sor, *Variations on a theme by Mozart—Theme*) was strongly associated with image no. 15 (“Matilada and Beatrice”). As an example, Figure 3 shows the scatterplot of the ratings given to the 22 adjectives for clip 3 (on the vertical axis) and image 5 (on the horizontal axis).

Most of the adjectives show a linear pattern, with low values for “happy,” “agitated,” “quick,” “presto,” and “strong” and high values for “sad,” “horizontal,” “slow,” “calm,” and “continuous.”

**Figure 4:**
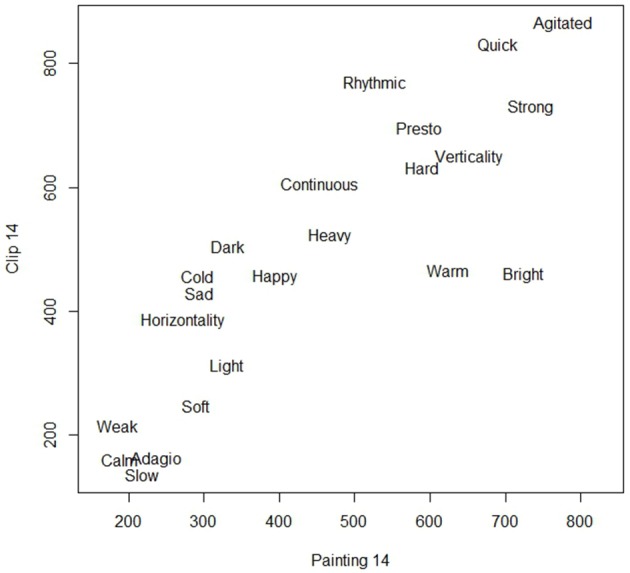
**Semantic rating of clip 14 (Villa Lobos, *Study n. 6*) and painting 14 (“Leopard”)**.

As a second example, Figure 4 shows the scatterplot of the ratings given to the 22 adjectives for clip 14 (on the vertical axis) and image 14 (on the horizontal axis).

Also in this case the adjectives show a linear pattern, with low values for “calm,” “slow,” “adagio,” and “weak,” and high values for “agitated,” “quick,” “strong,” and “presto.”

The study considered stimuli of great complexity. Consequently, we chose to have the maximum possible uniformity of style, composition and musical coloratura. The results show that the associations were made on specific characteristics that the subjects perceived as similar between the paintings and the music clips.

In particular, the associations between paintings and music clips proved to be consistent (even within the triads of images selected in the associations). Specifically, a strong positive association was found between clip no. 4 (Isaac Albéniz, *Asturias*) and image no. 14 (“Leopard”); between clip no. 14 (Villa Lobos, *Study n. 6*) and image no. 14 (“Leopard”); between clip no. 1 (Villa Lobos, *Prelude n. 4*) and image no. 10 (“Sea II”). To be noted is that the tempo of the first two music clips was either presto or prestissimo, and the images associated with them showed high values for the adjectives “quick,” “agitated,” and “strong.” The strongest negative association was instead between clip no. 3 (moderate tempo) and image no. 14 (“Leopard”). Clip no. 3 was associated with characteristics such as “continuous,” “slow,” “calm,” while the image “Leopard” was associated with opposite characteristics such as “agitated,” “bright,” “quick,” and “presto.” Considering the results presented in Figures 3, 4, the attributes that seemed to play the most significant role in the associations obtained were “calm,” “agitated,” “slow,” “quick,” “strong,” “presto,” “adagio.” Consequently, relevant features in the association with paintings seem to be the timbre and the musical tempo, as shown by the positive associations between clip no. 3 (Francisco Tárrega, *Recuerdos de la Alhambra*) and painting no. 5 (“Full Moon”), and between clip no. 14 (Villa Lobos, *Study n. 6*) and painting no. 14 (“Leopard”); and by the negative association between clip no. 3 and painting no. 14.

Contrary to what was expected (Bresin, 2005), the results instead show that the musical mode usually related to feelings of happiness (major mode), or to feelings of sadness (minor mode), and the spatial orientation (vertical and horizontal) as expressed by the attributes tested with the semantic differential, did not play a significant role in the association. Finally, no substantial difference was apparent between expert and non-expert subjects. Because all the images had highly saturated colors, we did not test for a potential association between these color dimensions and major and minor modes, or slow and fast music (see Bresin, 2005; Palmer et al., 2013). The purpose of our study was not to analyse the production of visual representation of a sequence of sounds in simple drawings as in Küssner (2013b; see also Küssner, 2013a), but the association of highly complex paintings and musical pieces of classical music. The two studies are then only partially comparable because our goal was not the visualization of music. What we asked the subjects to do was associate highly complex Gestalten in the visual and acoustic fields (not single parameters such as pitch and loudness) while listening to the clips. In other words, the task was much more complex and closer to the natural global perception of stimuli in the environment (in this case, of an artistic kind). Also different from Küssner (2013b) was the expertise of the participants; in fact we tested experts in music, non-experts in music, and art experts, but obviously we did not test experts in dance because our goal was not to test the motor action aspects of the associations (see also Maes et al., 2014). As to the study conducted by Cowles (1935), there were differences in the number and the kind of stimuli, in the aims and in the methodology: in Cowles' test the pictures, as mentioned, were mainly landscapes or scenes with simple content, while there was greater uniformity in our stimuli as to the paintings (which were by the same artist, and in the same style, materic and expressionist) and the music (all our clips were taken from Spanish classical guitar music). The contents of the paintings, instead, were different. In our experiment, besides the cross-modal association between auditory and visual stimuli, we also made use of the semantic differential method. But similarly to Cowles, our results showed no difference between experts and non-experts. The methods used in the experiment (i.e., semantic differential and subjective judgements) corroborated the interpretation of the results as associations due to patterns of qualitative similarity present in stimuli of different sensory modalities and experienced as such by the subjects (Albertazzi et al., 2013, 2014). Also in this respect the methodology that we used differed from the standard ones: we did not rely on psychophysical methods, reaction times (as in Marks, 2004; Spence, 2011), and forced choice responses (Walker, 1987); and we obviously did not make use of computational technologies. Our aim was to remain as close as possible to the natural perception of auditory and visual items. As said, the tested adjectives very frequently exhibited a linear pattern in the association between the paintings and the music clips: for example, having low values for “happy,” “agitated,” “quick,” “presto,” and strong and high values for “sad,” “horizontal,” “slow,” “calm,” and “continuous.” On the basis of these findings, and the fact that we didn't find any difference between expert and non-expert subjects, the tested semantic connotations of the stimuli might be considered as affordances playing the role of *general semantic information clues*, which makes perfect sense in a framework of an *ecology of meaning*. It has been recently shown, for example, that subjects in the general population group natural shapes on the basis of certain visual qualitative characteristics: specifically, non-spiculed, non-holed, and flat shapes are experienced and classified as harmonic and static, while rounded shapes are classified as harmonic and dynamic, and elongated shapes as somewhat disharmonious and somewhat static (Albertazzi et al., 2014). Because of the complex nature of the stimuli, and on the basis of our results, one can conclude that there are aesthetic, sometimes ideaesthetic dimensions in perceptual awareness. These dimensions act as Gestalten or templates playing the role of an immediate understanding of the complex objects we usually encounter in the environment. Furthermore, these Gestalten exhibit common patterns in the different modalities, as we have found in our study.

Finally, in our study we did not specifically test the emotional response, as in Cowles (1935), Di Dio and Gallese (2009), Juslin and Sloboda (2001), Krumhansl and Lerdahl (2011), Langlois et al. (2014), Madison (2011), Palmer et al. (2013) and Zaidel (2010), because it was not our primary interest. However, some of the adjectives tested with the semantic differential test, such as “calm” and “agitated,” “happy” and “sad” proved to have an important role.

In light of the overall results, one cannot exclude the presence of potential top-down influences (however unconscious), although our study did not aim to investigate these aspects. In this regard, what we did in our experiments was to invite the subjects to be as careful as possible to avoid the influence of past experience.

On the basis of our results, it is likely that the choice of a different number of adjectives restricted to a small number of characteristics, and limiting the range of associations and the length of the experiment, might yield further consistent information about the cross-modal associations obtained. Presenting adjectives in pairs, like calm/agitated, weak/strong, might also contribute to shortening the duration of the test. However, such a choice would have overestimated the correlation which in our study is also possibly overestimated, because the adjectives were not entirely independent. It is also likely that choosing a more uniform theme for the paintings (only landscapes, for example) would make the test shorter. A further development of the design might consist in testing the associations between the paintings and a series of music clips from a different musical repertoire, reducing the uniformity of patterns. Nevertheless, it seems worthwhile to continue testing cross-modal associations in complex stimuli, because these are usually experienced in perceiving. Finally, it would be advisable to repeat the experiment with subjects from other cultures, such as oriental ones, in order to test for the presence of possible pictorial and musical biases in the associations found.

In conclusion, our study shows (i) the existence of cross-modal associations between complex visual and auditory stimuli, (ii) the existence of associations between visual and auditory stimuli when evaluated employing the semantic differential, and (iii) that these associations were at least partially consistent with each other. These findings corroborate the interpretation that the associations are partially due to patterns of qualitative similarity present in stimuli of different sensory modalities.

### Conflict of interest statement

The authors declare that the research was conducted in the absence of any commercial or financial relationships that could be construed as a potential conflict of interest.

## References

[B1] Albertazzi L. (2013). Experimental phenomenology. An introduction, in The Wiley Blackwell Handbook of Experimental Phenomenology. Visual Perception of Shape, Space and Appearance, ed Albertazzi L. (London: Wiley-Blackwell), 1–36 10.1002/9781118329016

[B2] Albertazzi L. Canal L. Dadam J. Micciolo R. (2014). The semantics of biological forms. Perception 43, 365–1376 10.1068/p7794 25669053

[B3] Albertazzi L. Canal L. Malfatti M. Micciolo R. (2015). The hue of angles. Was Kandinsky right? Art Percept. 3, 81–92 10.1163/22134913-00002025

[B4] Albertazzi L. Da Pos O. Canal L. Micciolo R. Malfatti M. Vescovi M. (2013). The hue of shapes. J. Exp. Psychol. Hum. Percept. Perform. 39, 37–47. 10.1037/a0028816 22708741

[B5] Baron-Cohen S. Harrison J. E. (1997). Synesthesia: Classic and Contemporary Readings. Cambridge, MA: Blackwell.

[B6] Bidaine P. (Ed.). (2004). Sons & Lumières. Une Histoire du son Dans l'art du XXe Siècle. Paris: Éditions du Centre Pompidou.

[B7] Bresin R. (2005). What is the color of that music performance?, in Proceedings of the International Computer Music Conference–ICMC 2005 (International Computer Music Association, San Francisco, CA.)

[B8] Canal L. Micciolo R. (2013). Measuring the immeasurable: quantitative analyses of perceptual experiments, in Handbook of Experimental Phenomenology. Visual Perception of Shape, Space and Appearances, ed Albertazzi L. (London: Wiley-Blackwell), 477–498 10.1002/9781118329016.ch20

[B10] Chen N. Tanaka K. Watanabe K. (2015). Color-shape associations revealed with implicit association tests. PLoS ONE 10:e0116954. 10.1371/journal.pone.0116954 25625717 PMC4308101

[B11] Cohen Kadosh R. C. Henik A. Walsh V. (2009). Synesthesia: learned or lost? Dev. Sci. 12, 484–491. 10.1111/j.1467-7687.2008.00798.x 19371373

[B12] Cowles J. T. (1935). An experimental study of the pairing of certain auditory and visual stimuli. J. Exp. Psychol. 18, 461–469 10.1037/h0062202

[B13] Cytowic R. E. (1995). Synesthesia: A Union of the Senses, 2nd Edn. Cambridge MA: MIT Press.

[B14] Dadam J. Albertazzi L. Da Pos O. Canal L. Micciolo R. (2012). Morphological patterns and their colours. Percept. Mot. Skills 114, 363–377. 10.2466/03.22.23.PMS.114.2.363-377 22755441

[B15] Demattè L. Sanabria D. Spence C. (2006). Cross-modal associations between odors and colours. Chem. Senses 31, 531–538. 10.1093/chemse/bjj057 16648449

[B16] Deroy O. Crisinel A. Spence C. (2013). Crossmodal correspondences between odours and contingent features: odours, musical notes and abstract shapes. Psychon. Bull. Rev. 20, 1–19. 10.3758/s13423-013-0397-0 23463615

[B17] Deroy O. Spence C. (2013). Why we are not all synesthetes (not even weakly so). Psychon. Bull. Rev. 20, 643–664. 10.3758/s13423-013-0387-2 23413012

[B18] Di Dio C. Gallese V. (2009). Neuroaesthetics: a review. Curr. Opin. Neurobiol. 19, 682–687. 10.1016/j.conb.2009.09.001 19828312

[B19] Dixon M. J. Smilek D. Duffy P. L. Zanna M. P. Merikle P. M. (2006). The role of meaning in grapheme-colour synesthesia. Cortex 42, 243–252. 10.1016/S0010-9452(08)70349-6 16683498

[B20] Droste M. (1990). Bauhaus. Köln: Taschen.

[B21] Eagleman D. M. (2010). Synaesthesia. Br. Med. J. 340, b4616. 10.1136/bmj.b4616 20061356

[B22] Eagleman D. M. (2012). Synesthesia in its protean guises. Br. J. Psychol. 103, 16–19. 10.1111/j.2044-8295.2011.02020.x 22229769

[B23] Gage J. (1999). Color and Meaning: Art, Science and Symbolism. Berkeley, CA: University of California Press.

[B24] Gallace A. Boschin E. Spence C. (2011). On the taste of “bouba” and “kiki”: an exploration of word-food associations in neurologically normal participants. Cogn. Neurosci. 2, 34–46. 10.1080/17588928.2010.516820 24168422

[B25] Gilbert A. N. Martin R. Kemp S. E. (1996). Cross-modal correspondence between vision and olfaction: the color of smells. Am. J. Psychol. 109, 335–351. 8837406

[B26] Hanson-Vaux G. Crisinel A.-S. Spence C. (2013). Smelling shapes: crossmodal correspondences between odors and shapes. Chem. Senses 38, 161–166. 10.1093/chemse/bjs087 23118203

[B27] Itten J. Birren F. van Haagen E. (1970). The Elements of Color: A Treatise on the Color System of Johannes Itten Based on his Book The Art of Color. New York, NY: Van Nostrand Reinhold.

[B28] Jacobsen T. H. (2002). Kandinsky's questionnaire revisited: fundamental correspondence of basic colors and forms? Percept. Mot. Skills 95, 903–913. 10.2466/pms.2002.95.3.903 12509195

[B29] Jürgens U. M. Nikolić D. (2012). Ideaesthesia: conceptual processes assign similar colours to similar shapes. Translat. Neurosci. 3, 22–27 10.2478/s13380-012-0010-4

[B30] Jürgens U. M. Nikolić D. (2014). Synesthesia as an Ideasthesia - cognitive implications, in Synesthesia and Children-Learning and Creativity, eds Sinha J. R. Söffing C. (Kassel: Kassel University Press), (in press).

[B31] Juslin P. N. Sloboda J. A. (2001). Music and Emotion: Theory and Research. Oxford: Oxford University Press.

[B32] Kandinsky W. (1926/1994). Point and line to plane (P. Vergo, Trans.), in Kandinsky, Complete Writings on Art, eds Lindsay K. C. Vergo P. (New York, NY: Da Capo Press), 524–700.

[B33] Kemp S. E. Gilbert A. N. (1997). Odor intensity and color lightness are correlated sensory dimensions. Am. J. Psychol. 110, 35–46. 10.2307/1423699 9100340

[B34] Kharkhurin A. V. (2012). Is triangle really yellow? An empirical investigation of Kandinsky's correspondence theory. Empir. Stud. Arts 30, 167–182 10.2190/EM.30.2.d

[B35] Klee P. (1956). Das bildnerische Denken. Schriften zur Form und Gestaltungslehre. Basel-Stuttgart: Jürg Spiller editor.

[B36] Köhler W. (1929). Gestalt Psychology. New York, NY: Liveright.

[B37] Krumhansl C. H. Lerdahl F. (2011). Musical tension, in Art and the Senses, eds Bacci F. Melcher D. (Oxford: Oxford University Press), 311–328.

[B38] Küssner M. B. (2013a). Shaping music visually, in Kreativität – Struktur und Emotion, eds Lehmann A. C. Jeßulat A. Wünsch C. (Würzburg: Königshausen & Neumann), 203–209.

[B39] Küssner M. B. (2013b). Music and shape. Lit. Linguist. Comput. 28, 472–479 10.1093/llc/fqs071

[B40] Langlois T. Peterson J. Palmer S. (2014). Visual Texture, Music and Emotion. Abstract presented at the 13th Annual Meeting of the Vision Sciences Society (St. Pete Beach, FL).

[B41] Levitan C. A. In Ren J. Woods A. Boesveldt S. Chan J. S. McKenzie J.. (2014). Cross-cultural color-odor associations. PLoS ONE 9:e101651. 10.1371/journal.pone.0101651 25007343 PMC4089998

[B42] Ludwig V. U. Simner J. (2013). What colour does that feel? Tactile-visual mapping and the development of cross-modality. Cortex 59, 1089–1099. 10.1016/j.cortex.2012.04.004 22622436

[B43] Lupton E. Miller J. A. (1991). The ABC's of Design: The Bauhaus and Design Theory. New York, NY: Princeton Architectural Press.

[B44] Madison G. (2011). Cause and effect: a functional perspective on music and emotion, in Art and the Senses, eds Bacci F. Melcher D. (New York, NY: Oxford University Press), 329–350.

[B45] Maes P.-J. Leman M. Palmer C. Wanderley M. M. (2014). Action-based effects on music perception. Front. Psychol. 4:1008 10.3389/fpsyg.2013.01008 24454299 PMC3879531

[B46] Makin A. D. J. Wuerger S. (2013). The IAT shows no evidence for Kandinsky's color-shape associations. Front. Psychol. 4:616. 10.3389/fpsyg.2013.00616 24062709 PMC3769683

[B47] Marks L. E. (2004). Cross-modal interactions in speeded classification, in Cross-Modal Interactions in Speeded Classification, eds Calvert G. A. Spence C. Stein B. E. (Cambridge, MA: MIT Press), 85–105.

[B48] Martino G. Marks L. E. (2001). Synesthesia: strong and weak. Curr. Dir. Psychol. Sci. 10, 61–65 10.1111/1467-8721.00116

[B49] Maurer D. Mondloch C. (2005). Neonatal synesthesia: A revaluation, in Perspective from Cognitive Neuroscience, eds Robertson L. Sagiv N. (Oxford: Oxford University Press), 193–213.

[B50] Meier B. (2013). Semantic representation of synesthesia. Theor. Hist. Sci. 10, 125–134 10.12775/ths-2013-0006

[B51] Melara R. D. O'Brien T. P. (1987). Interactions between synesthetically corresponding dimensions. J. Exp. Psychol. Gen. 116, 323–336. 10.1371/journal.pone.0005664 19471644 PMC2680950

[B52] Moos A. Simmons D. Simner J. Smith R. (2013). Color and texture associations in voice-induced synaesthesia. Front. Psychol. 4:568. 10.3389/fpsyg.2013.00568 24032023 PMC3759022

[B53] Moos A. Smith R. Miller S. R. Simmons D. P. (2014). Cross-modal associations in synesthesia: vowel colours in the ear of the beholder. i-perception 5, 132–142. 10.1068/i0626 25469218 PMC4249992

[B54] Mroczko-Wąsowicz A. Nikolić D. (2014). Semantic mechanisms may be responsible for developing synesthesia. Front. Hum. Neurosci. 8:509. 10.3389/fnhum.2014.00509 25191239 PMC4137691

[B55] Mroczko-Wąsowicz A. Werning M. (2012). Synesthesia, sensory-motor contingency, and semantic emulation: how swimming style-color synesthesia challenges the traditional view of synesthesia. Front. Psychol. 3:279 10.3389/fpsyg.2012.00279 PMC3425383 22936919

[B56] Nielsen A. Rendall D. (2011). The sound of round: evaluating the sound-symbolic role of consonants in the classic Takete-Maluma phenomenon. Can. J. Exp. Psychol. 65, 115–124. 10.1037/a0022268 21668094

[B57] Osgood C. E. (1956). Method and Theory in Experimental Psychology. Oxford: Oxford University Press.

[B58] Palmer S. E. Schloss K. B. (2010). An ecological valence theory of color preferences. Proc. Natl. Acad. Sci. U.S.A. 107, 8877–8882. 10.1073/pnas.0906172107 20421475 PMC2889342

[B59] Palmer S. E. Schloss K. B. Xu Z. Prado-León L. R. (2013). Music-colour associations are mediated by emotion. Proc. Natl. Acad. Sci. U.S.A. 110, 8836–8841. 10.1073/pnas.1212562110 23671106 PMC3670360

[B60] Parise C. Spence C. (2009). ‘When birds of a feather flock together’: synesthetic correspondences modulate audiovisual integration in non-synesthetes. PLoS ONE 4:e5664. 10.1371/journal.pone.0005664 19471644 PMC2680950

[B61] R. Core Team (2013). R: A Language and Environment for Statistical Computing. Vienna: R Foundation for Statistical Computing.

[B62] Reybrouck M. Verschaffel L. Lauwerier S. (2009). Children's graphical notations as representational tools for musical sense-making in a music-listening task. Br. J. Music Educ. 26, 189–211 10.1017/S0265051709008432

[B64] Sagiv N. Simner J. Collins J. Butterworth B. Ward J. (2006). What is the relationship between synaesthesia and visuo-spatial number forms? Cognition 101, 114–128. 10.1016/j.cognition.2005.09.004 16288733

[B65] Sagiv N. Ward J. (2006). Crossmodal interactions: lessons from synesthesia. Prog. Brain Res. 155, 259–271. 10.1016/S0079-6123(06)55015-0 17027393

[B66] Schönberg A. Kandinsky W. (1980). Briefe, Bilder und Dokumente einer aussergewöhnlichen Begegnung. St. Pölten: Residenz Verlag.

[B67] Simner J. (2012). Defining synaesthesia. Br. J. Psychol. 103, 1–15. 10.1348/000712610X528305 22229768

[B68] Simner J. Gartner O. Taylor M. D. (2011). Cross-modal personality attributions in synaesthetes and non-synaestetes. J. Neuropsychol. 5, 283–301. 10.1111/j.1748-6653.2011.02009.x 21923790

[B69] Simner J. Lanz M. Jansari A. Noonan K. Glover L. Oakley D. A.. (2005). Nonrandom associations of graphemes to colours in synaesthetic and normal populations. Cogn. Neuropsychol. 22, 1069–1085. 10.1080/02643290500200122 21038290

[B70] Simner J. Mulvenna C. Sagiv N. Tsakanikos E. Witherby S. A. Fraser C. (2006). Synaesthesia: the prevalence of atypical cross-modal experiences. Perception 35, 1024–1033. 10.1068/p5469 17076063

[B71] Simner J. Ward J. (2006). Synesthesia: the taste of words on the tip of the tongue. Nature 444:438. 10.1038/444438a 17122848

[B72] Spector F. Maurer D. (2008). The colour of Os: naturally biased associations between shape and colour. Perception 37, 841–847. 10.1068/p5830 18686703

[B73] Spector F. Maurer D. (2011). The colours of the alphabet: naturally biased associations between shape and colour. J. Exp. Psychol. Hum. Percept. Perform. 37, 484–495. 10.1037/a0021437 21261419

[B74] Spence C. (2011). Crossmodal correspondences: a tutorial review. Atten. Percept. Psychophys. 73, 971–995. 10.3758/s13414-010-0073-7 21264748

[B75] Tan S. Kelly M. (2004). Graphic representations of short musical compositions. Psychol. Music 32, 191 10.1177/0305735604041494

[B76] Tomson S. N. Narayan M. Allen G. I. Eagleman D. (2013). Neural networks of colored sequence synesthesia. J. Neurosci. 33, 14098–14106. 10.1523/JNEUROSCI.5131-12.2013 23986245 PMC4050198

[B77] Walker R. (1987). The effects of culture, environment, age, and musical training on choices of visual metaphors for sound. Percept. Psychophys. 42, 491–502. 10.3758/BF03209757 2447557

[B78] Ward J. (2013). Synesthesia. Annu. Rev. Psychol. 64, 49–75. 10.1146/annurev-psych-113011-143840 22747246

[B79] Ward J. Huckstep B. Tsakanikos E. (2006a). Sound-colour synaesthesia: to what extent does it use cross-modal mechanisms common to us all? Cortex 42, 264–280. 10.1016/S0010-9452(08)70352-6 16683501

[B80] Ward J. Li R. Salih S. Sagiv N. (2007). Varieties of grapheme-colour synaesthesia: a new theory of phenomenological and behavioural differences. Conscious. Cogn. 16, 913–931. 10.1016/j.concog.2006.09.012 17126034

[B81] Ward J. Simner J. (2003). Lexical-gustatory synaesthesia. Linguistic and conceptual factors. Cognition 89, 237–261. 10.1016/S0010-0277(03)00122-7 12963263

[B82] Ward J. Tsakanikos E. Bray A. (2006b). Synaesthesia for reading and playing musical notes. Neurocase 12, 27–34. 10.1080/13554790500473672 16517513

[B83] Whiteford K. Schloss K. B. Palmer S. E. (2013). Music-Color associations from Bach to the Blues: Emotional mediation in synesthetes and non-synesthetes, in Poster Presented at the 13th Annual Meeting of the Vision Science Society (Neaples).

[B84] Wicker F. W. (1968). Mapping the intersensory regions of perceptual space. Am. J. Psychol. 81, 178–188. 10.2307/1421262 5747961

[B85] Zaidel D. W. (2010). Art and brain: insights from neuropsychology, biology and evolution. J. Anat. 216, 177–183. 10.1111/j.1469-7580.2009.01099.x 19490399 PMC2815940

