# Supplementary figures and images for: Cross-modal associations between materic painting and classical Spanish music
Source: Front Psychol. 2015 Apr 21;6:424. doi: 10.3389/fpsyg.2015.00424 (PMC4404723; doi:10.3389/fpsyg.2015.00424)

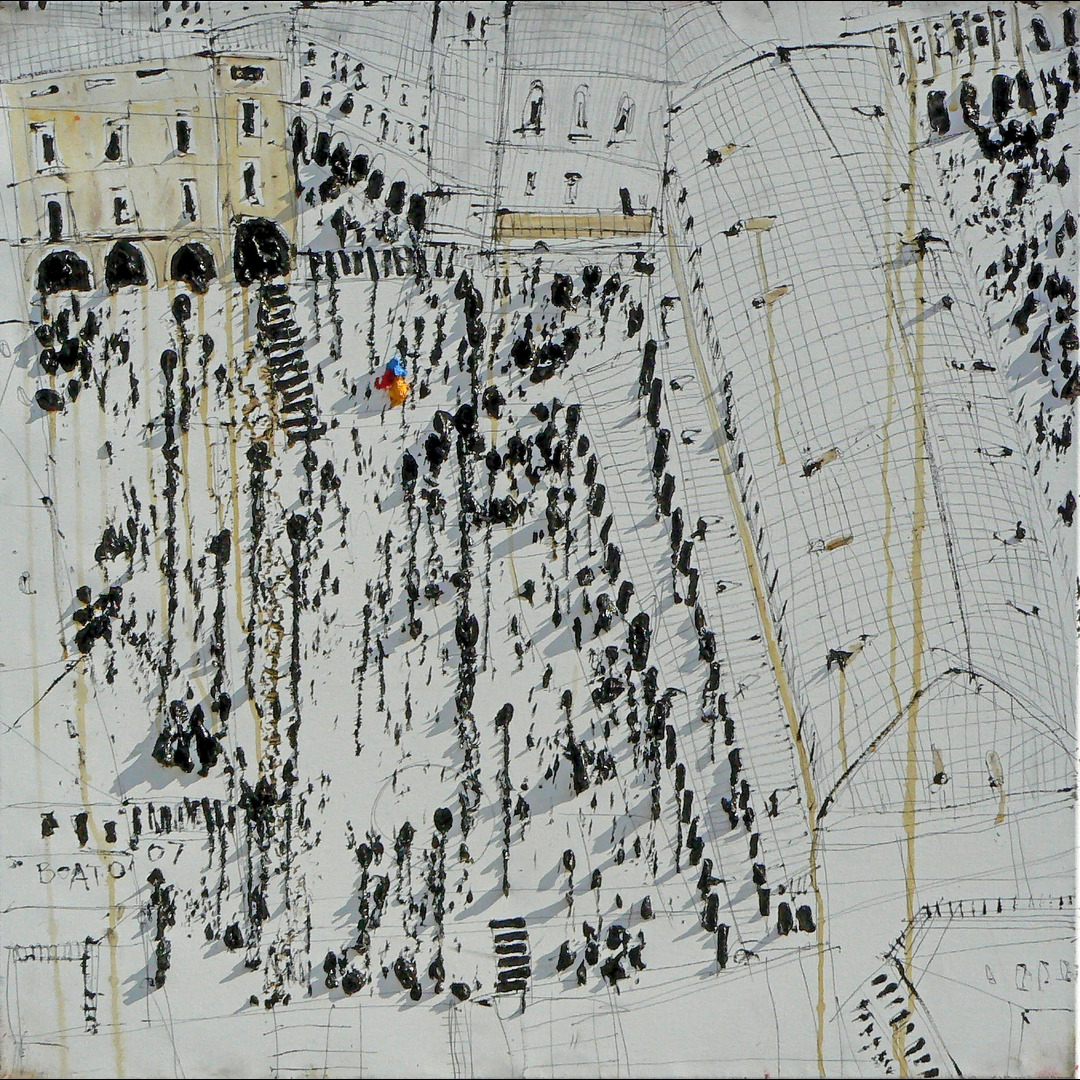

Supplement: Supplementary file 2 [file Presentation2.ZIP › 01 Padova,2007,80x80-A-1080.jpg]

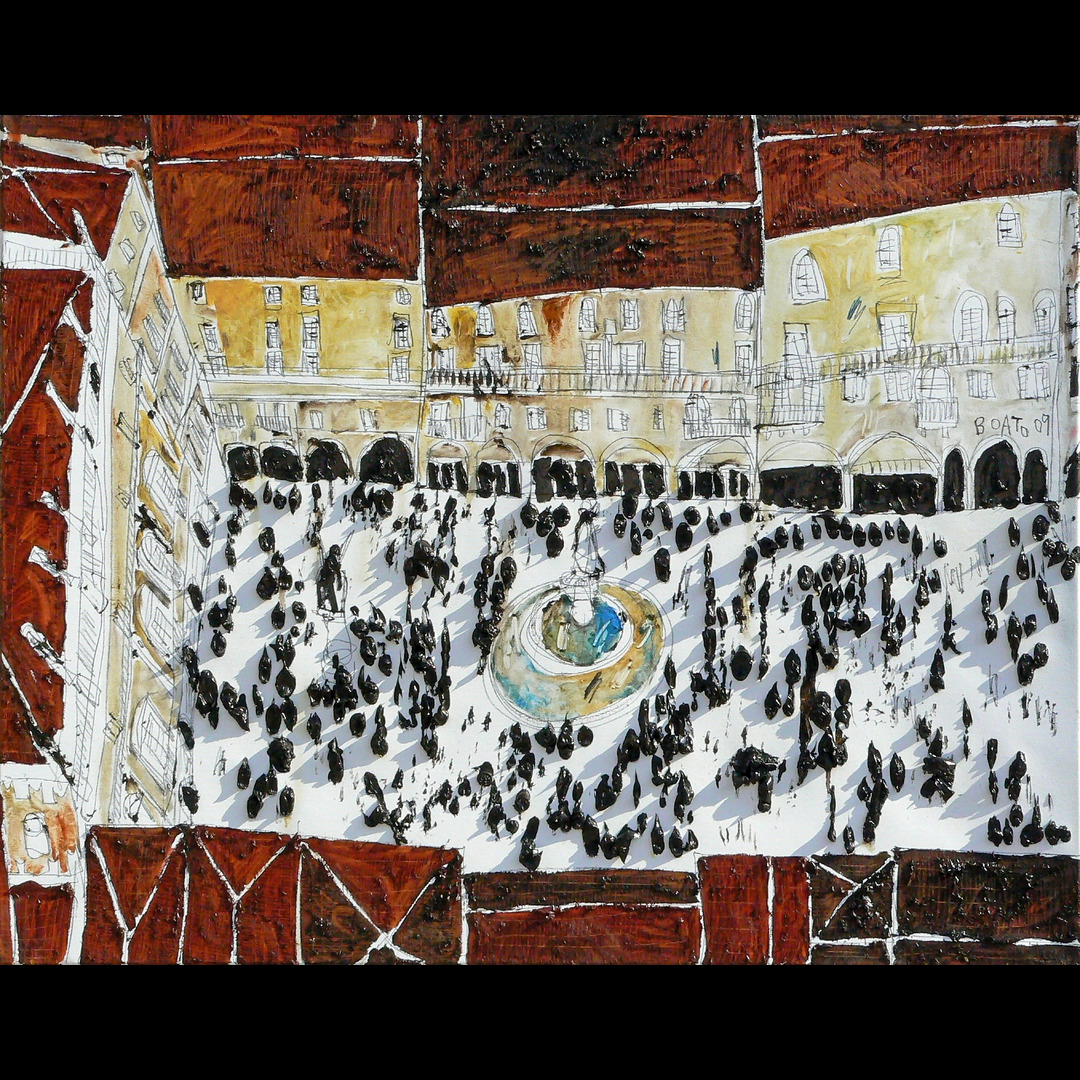

Supplement: Supplementary file 2 [file Presentation2.ZIP › 02 Verona,2009,olio su tela, 100x80-A-1080.jpg]

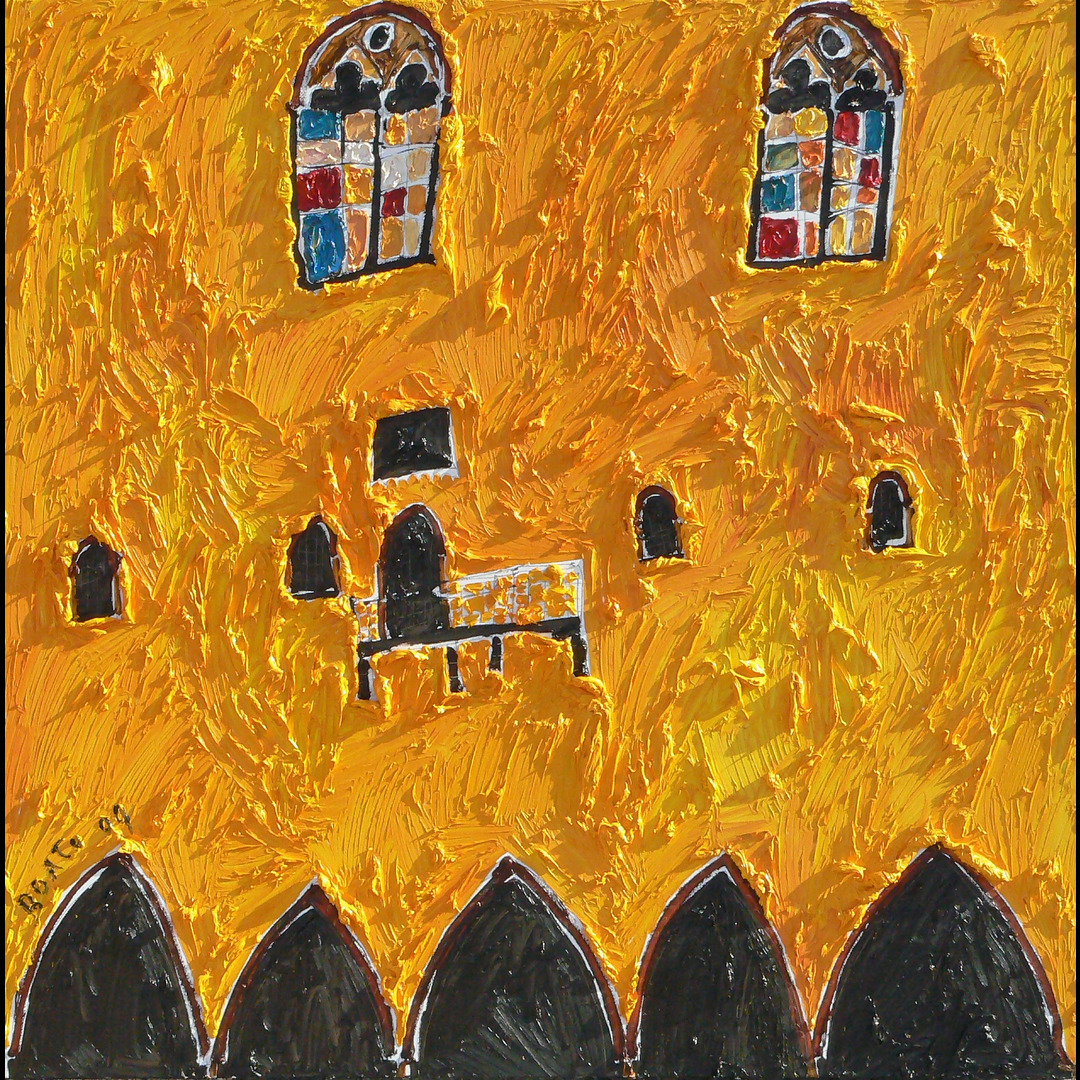

Supplement: Supplementary file 2 [file Presentation2.ZIP › 03 Mantova,2009,olio su tela, 90x90-A-1080.jpg]

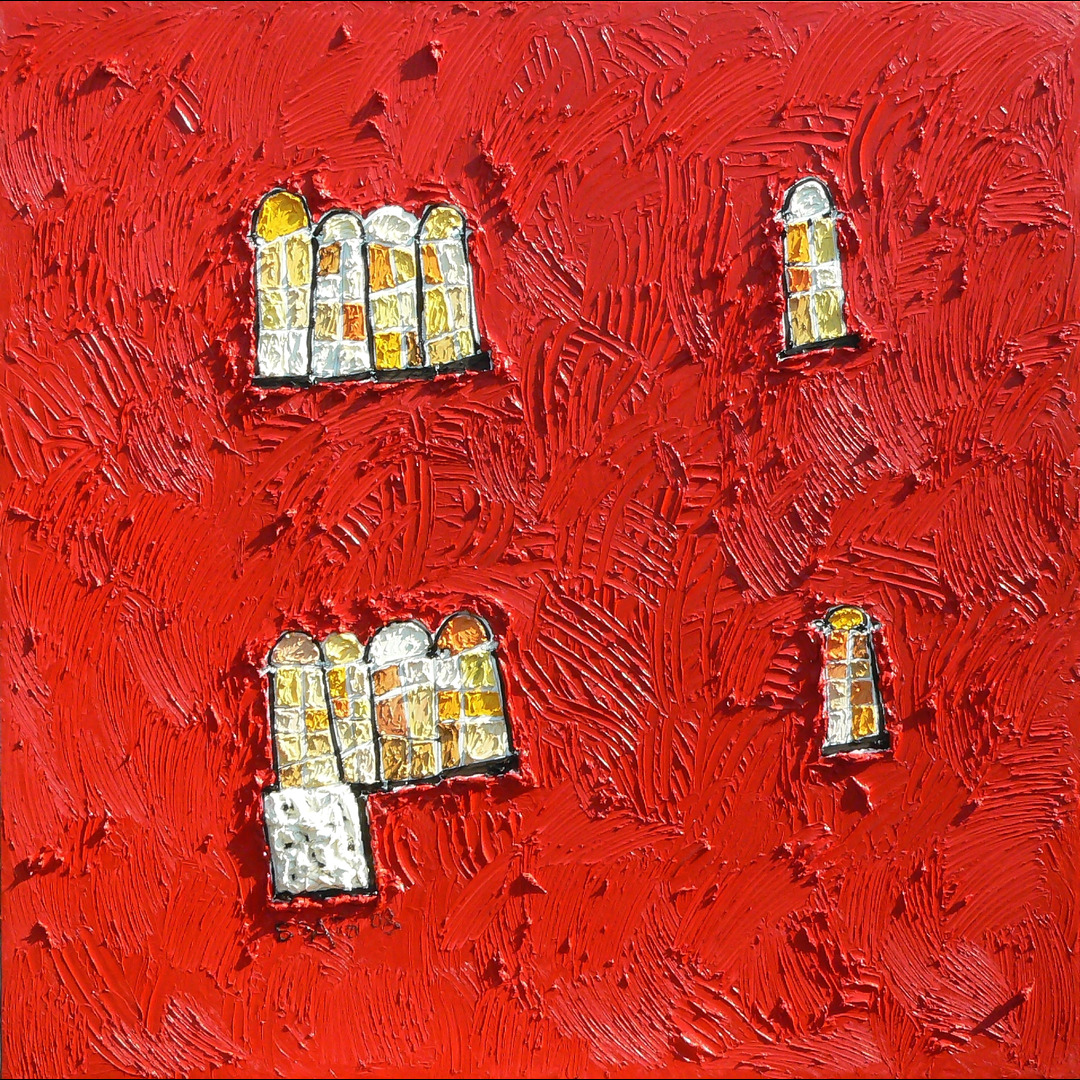

Supplement: Supplementary file 2 [file Presentation2.ZIP › 04 Trento, 2008,olio su tela, 150x150-A-1080.jpg]

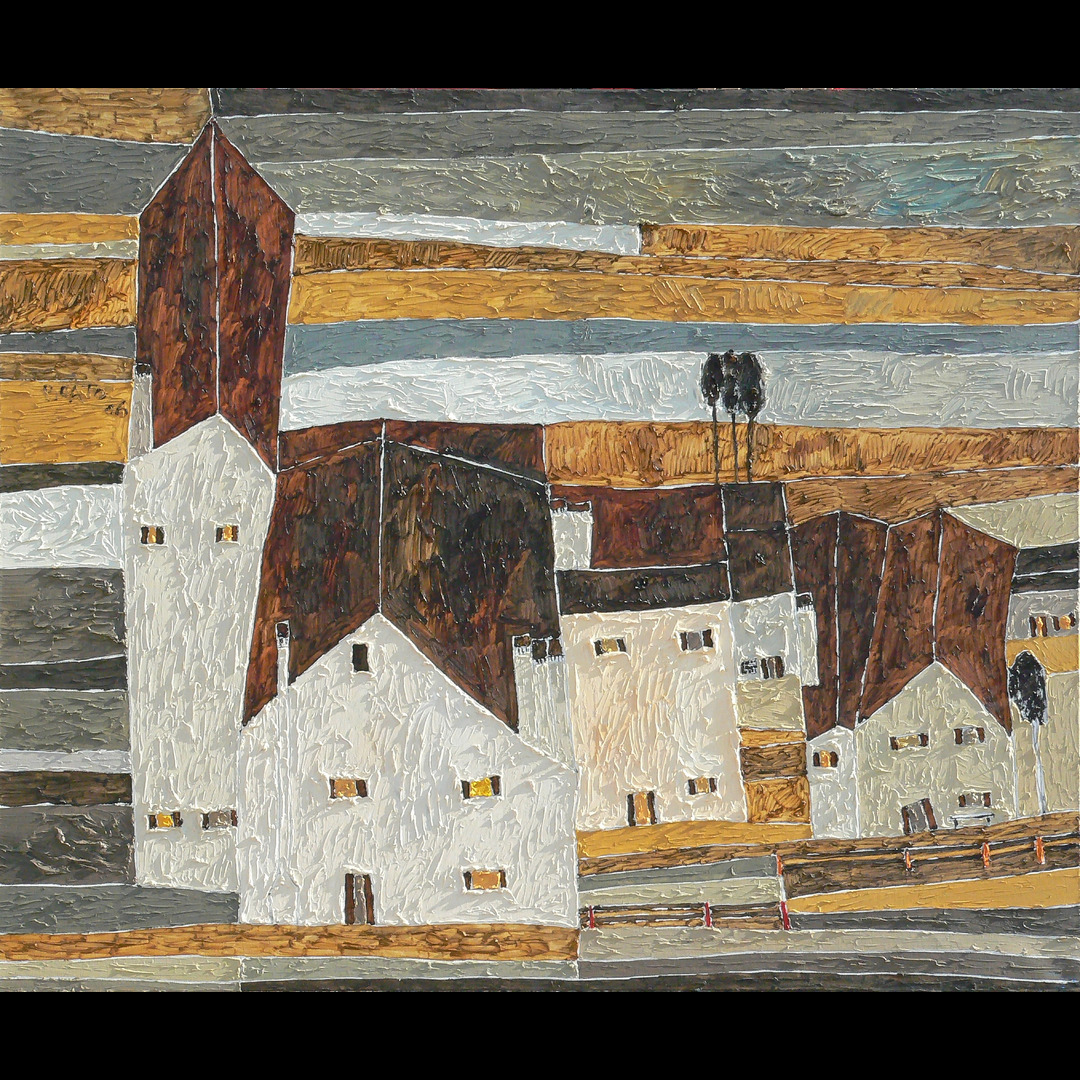

Supplement: Supplementary file 2 [file Presentation2.ZIP › 05 Plenilunio - dittico,2006,olio su tela, 120x100-A-1080.jpg]

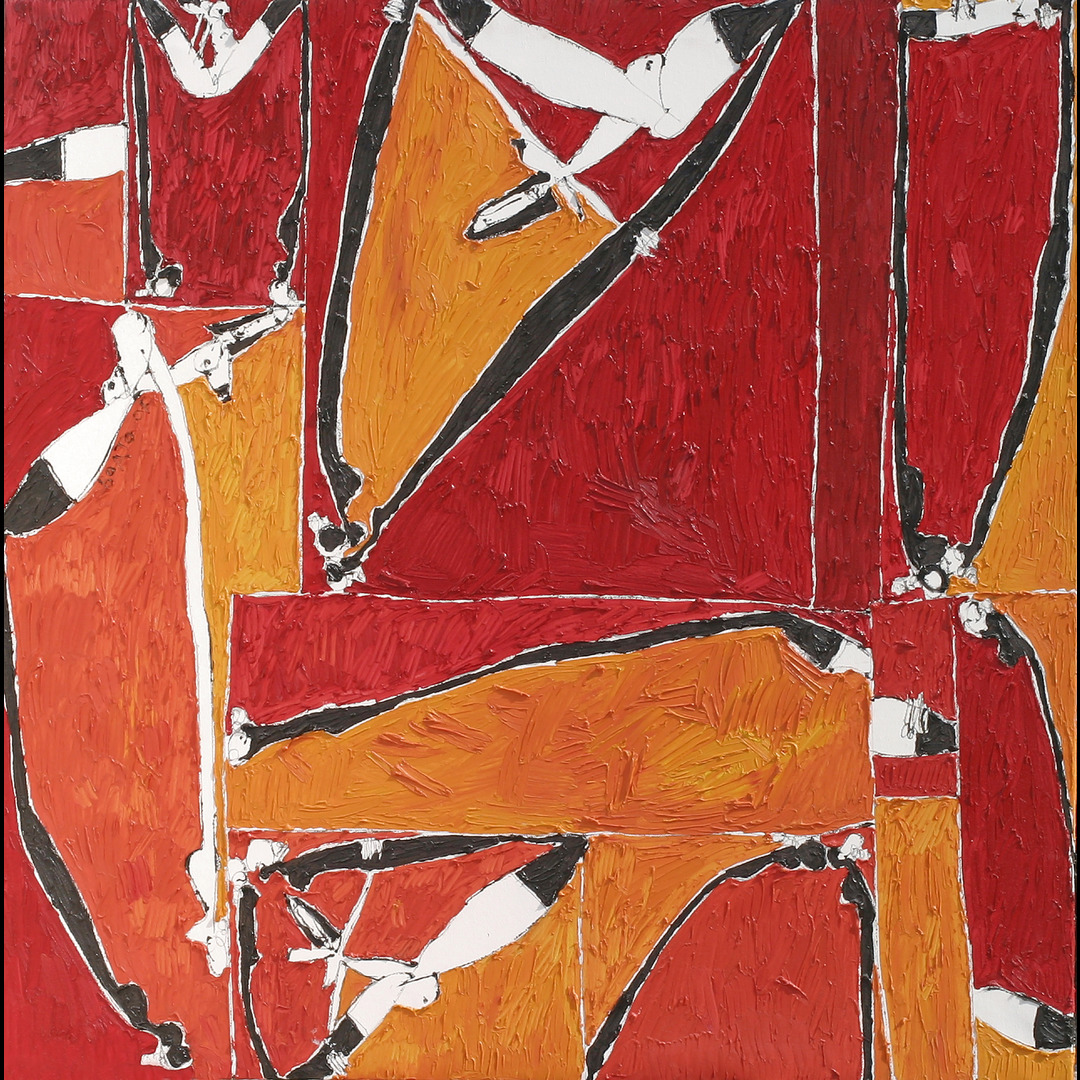

Supplement: Supplementary file 2 [file Presentation2.ZIP › 06 Il Cerchio, 100 x 100, olio su tela-A-1080.jpg]

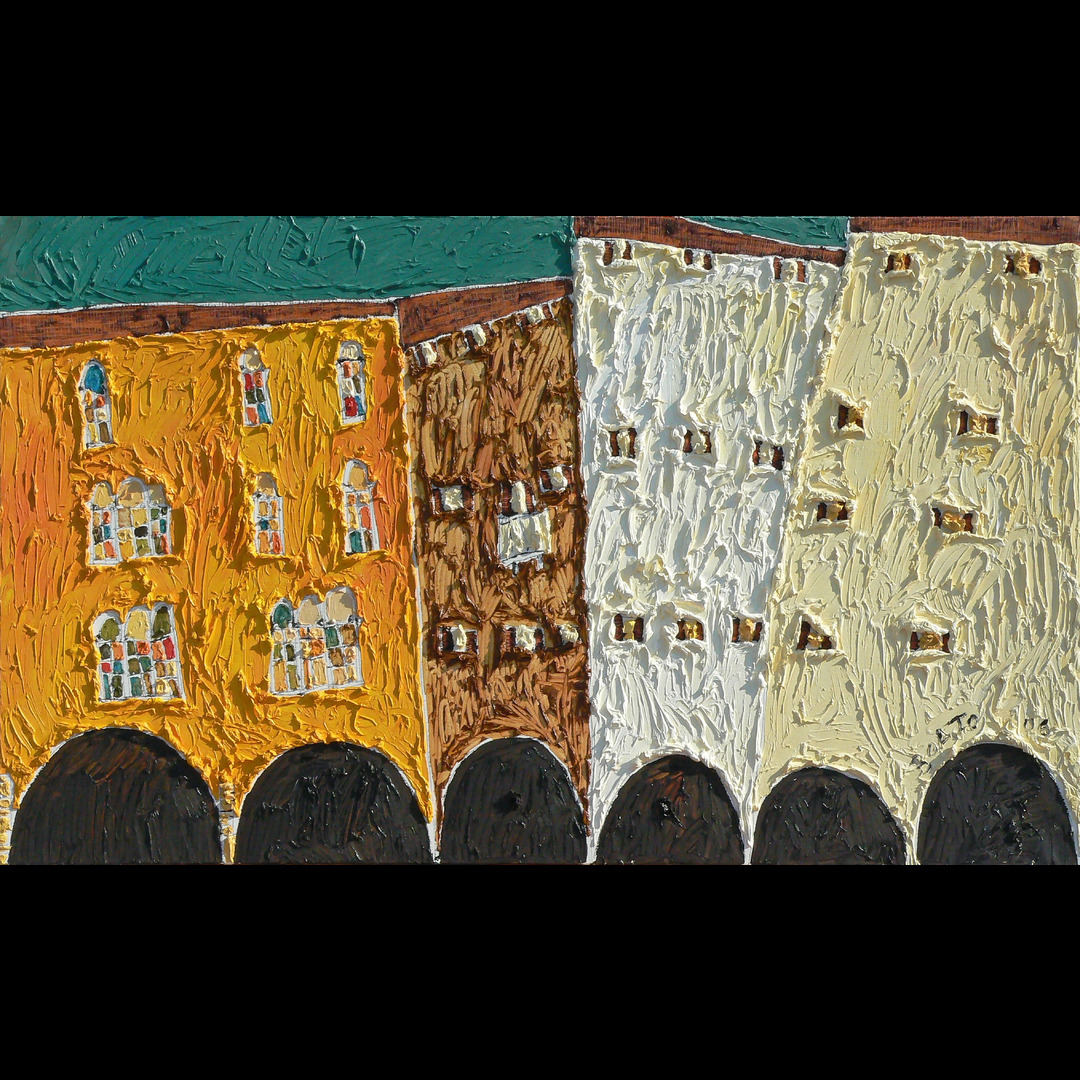

Supplement: Supplementary file 2 [file Presentation2.ZIP › 07 Trento,2006,olio su tela, 100x60-A-1080.jpg]

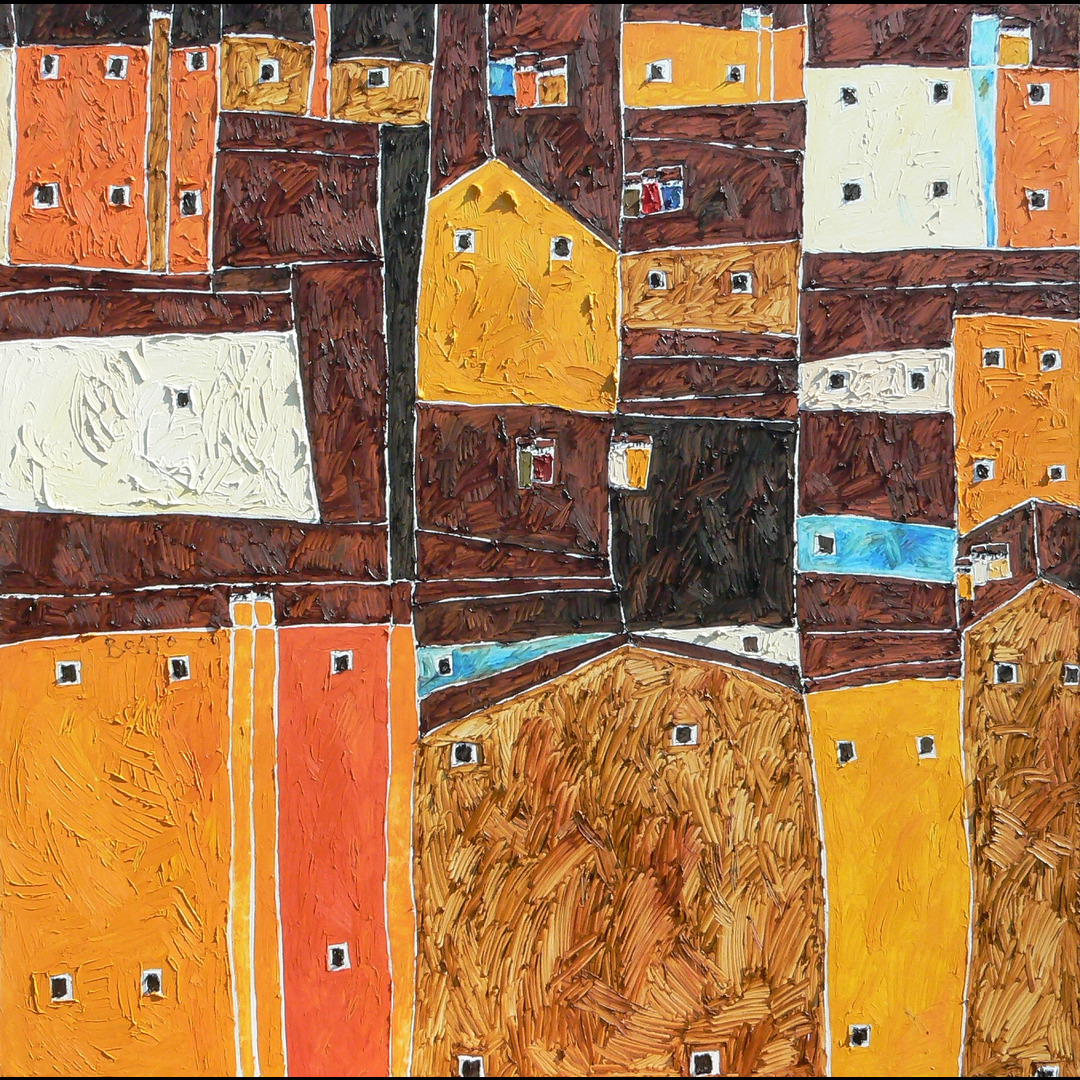

Supplement: Supplementary file 2 [file Presentation2.ZIP › 08 Burano, 2009,olio su tela, 150x150-A-1080.jpg]

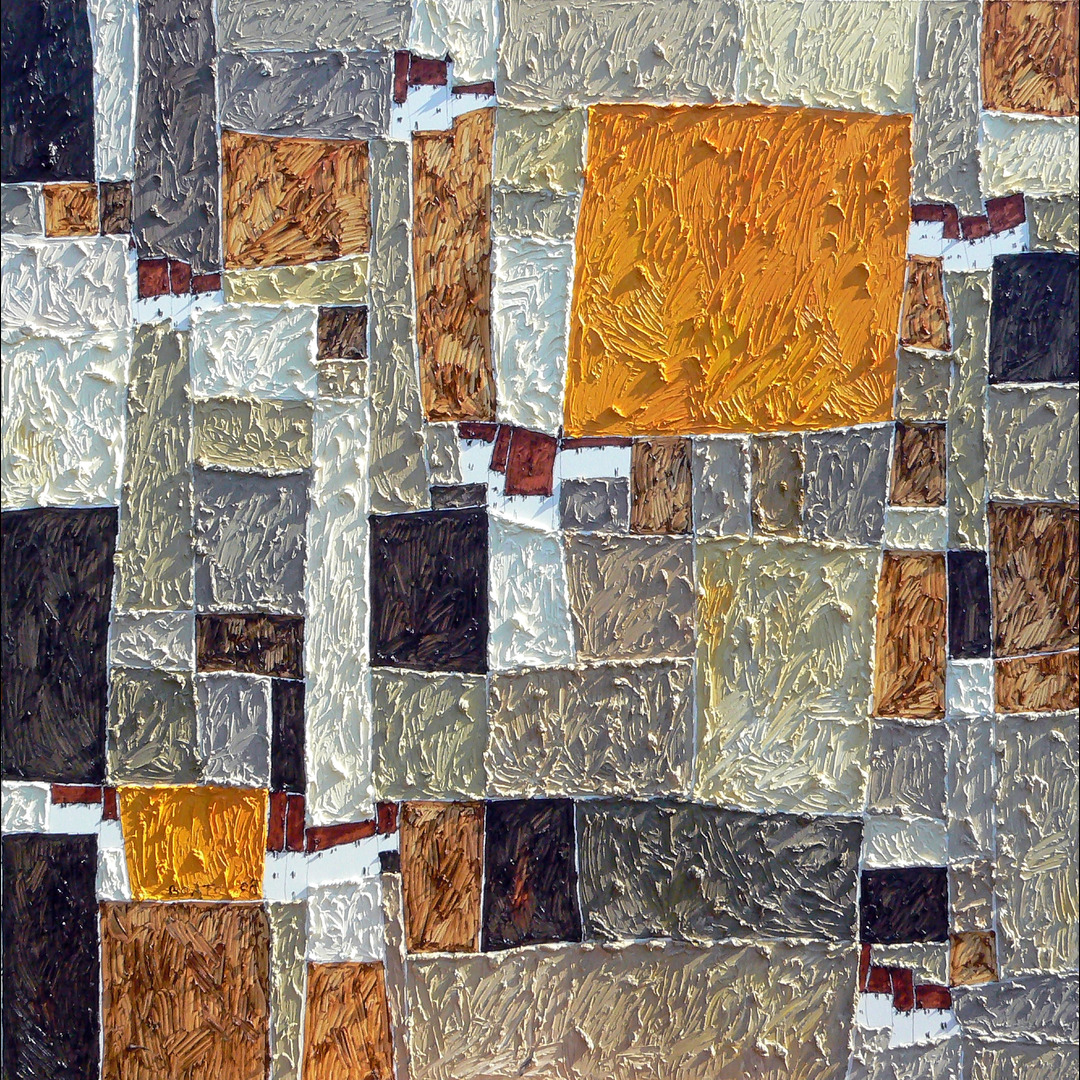

Supplement: Supplementary file 2 [file Presentation2.ZIP › 09 Cielo di Campi, 2009,olio su tela, 150x150-A-1080.jpg]

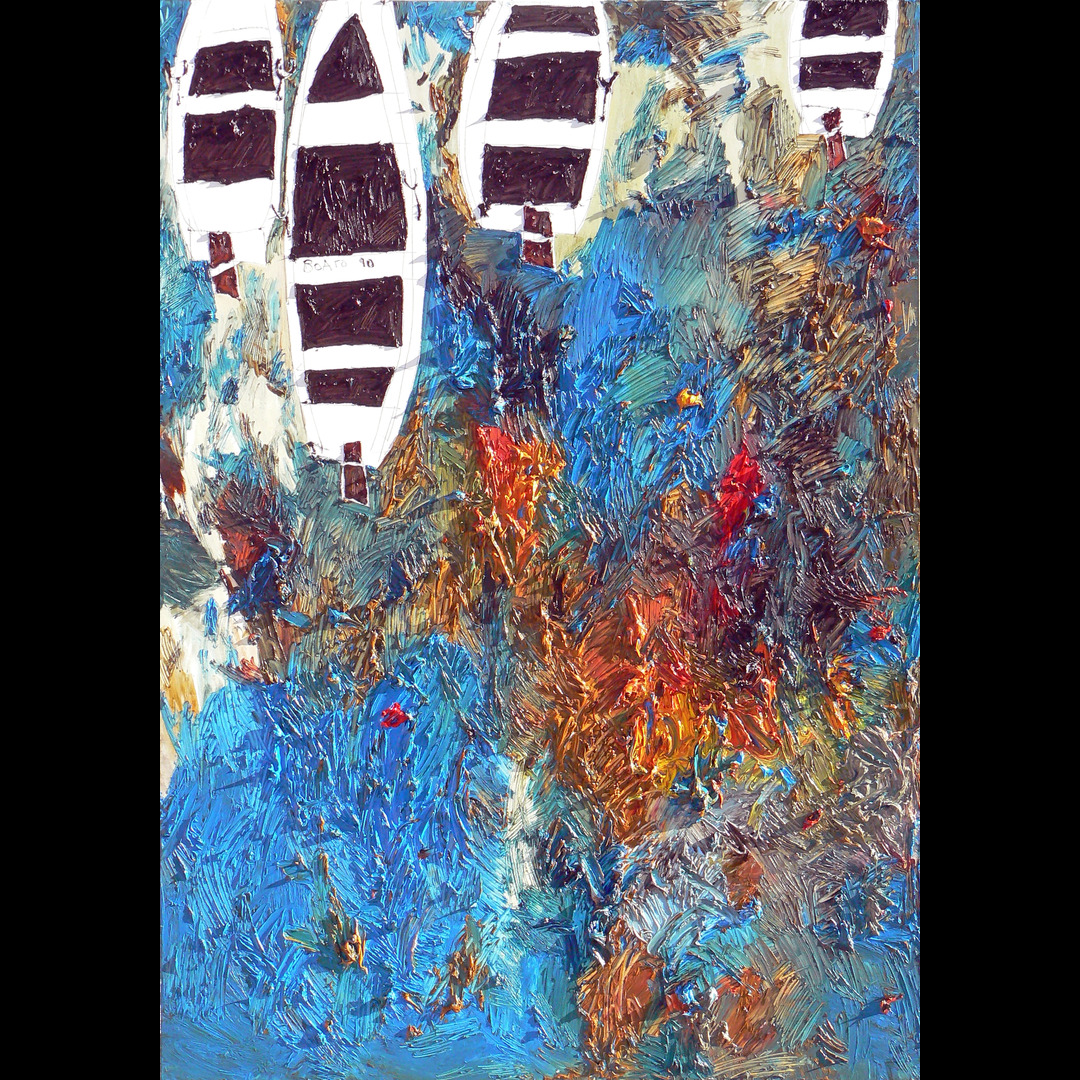

Supplement: Supplementary file 2 [file Presentation2.ZIP › 10 Mare II, 2010, olio su tela, 100x140-A-1080.jpg]

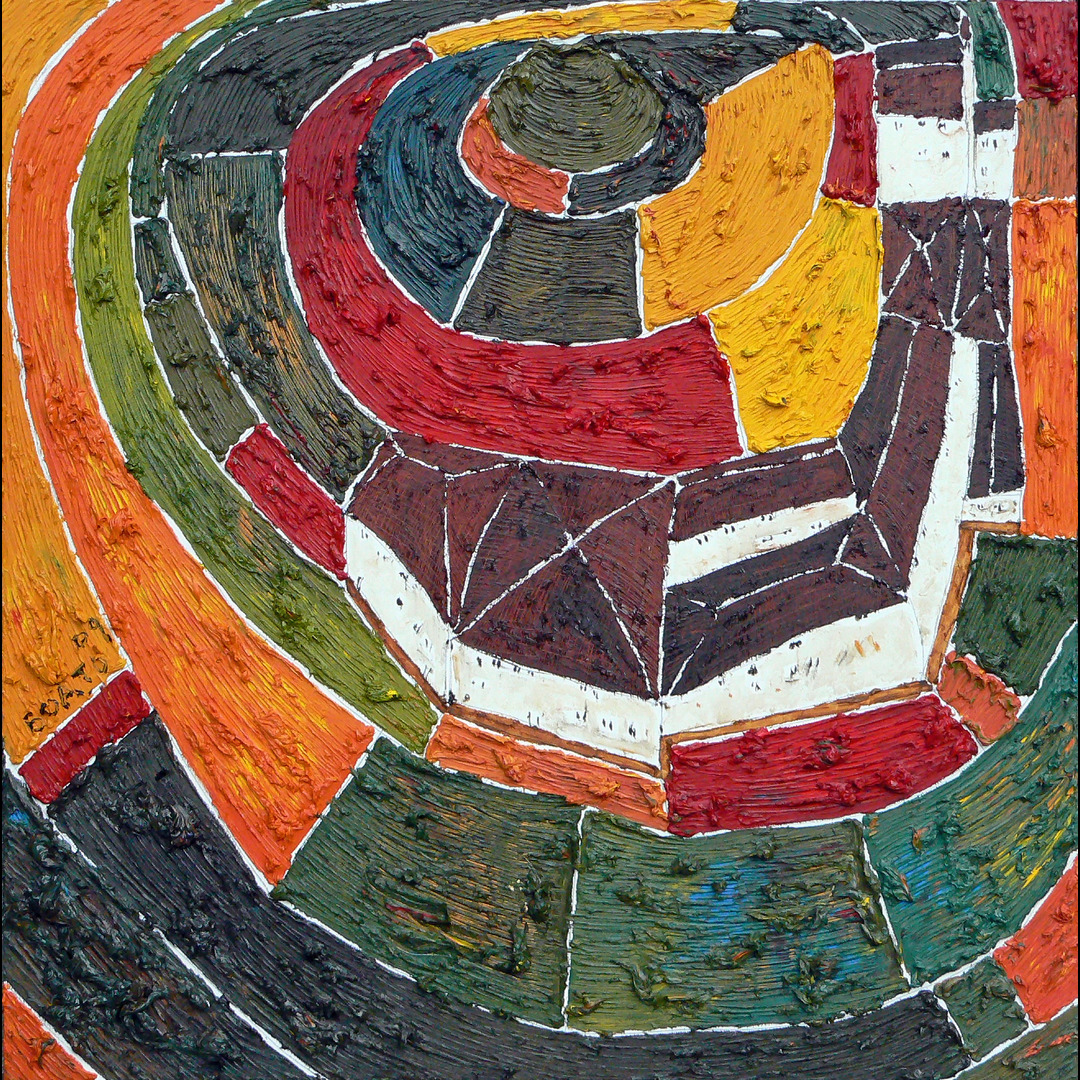

Supplement: Supplementary file 2 [file Presentation2.ZIP › 11 Terra II - Hora et Labora, 100x100 , olio su tela, Matteo Boato, 2009-A-1080.jpg]

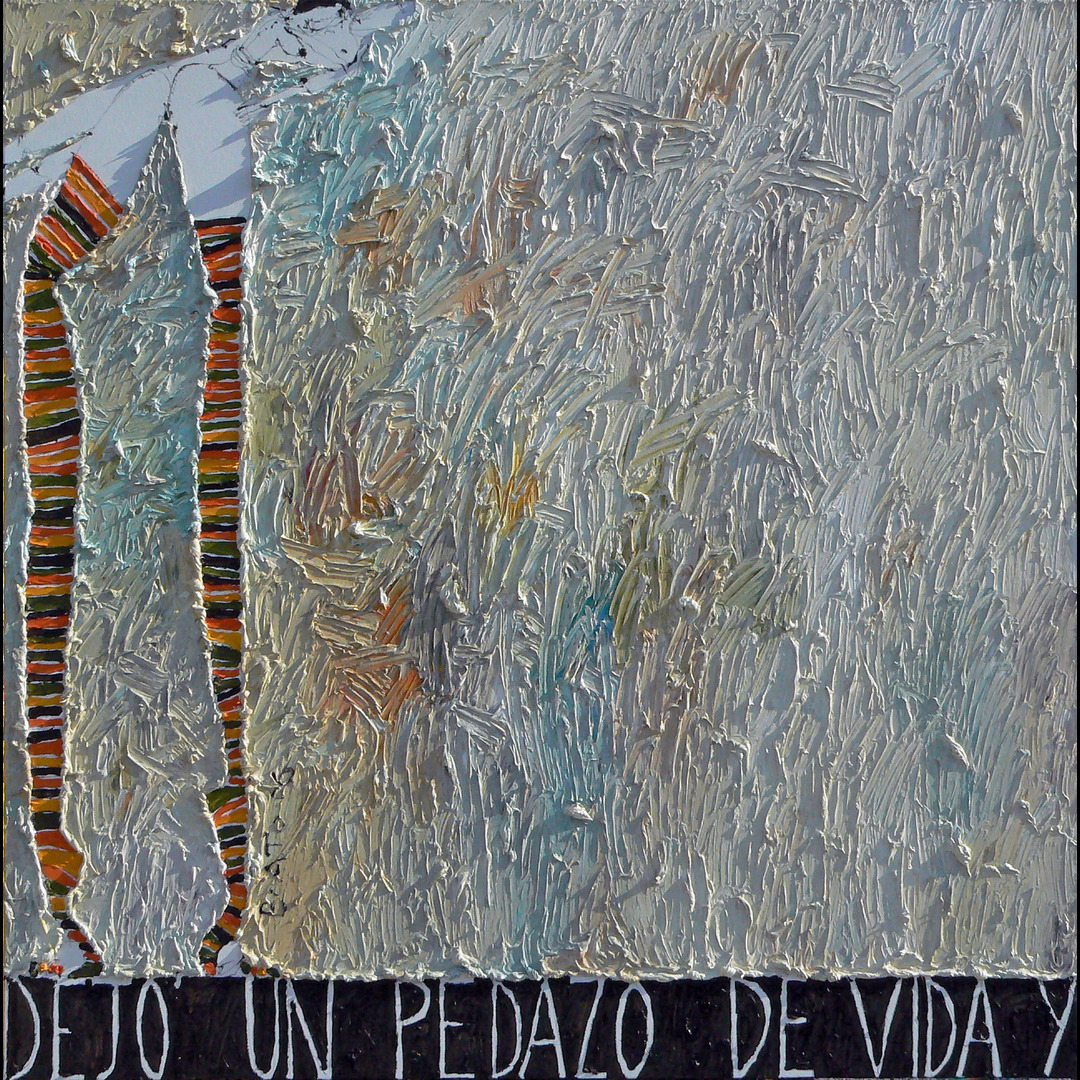

Supplement: Supplementary file 2 [file Presentation2.ZIP › 12 Insogno II, 100 x 100, olio su tela, 2006-A-1080.jpg]

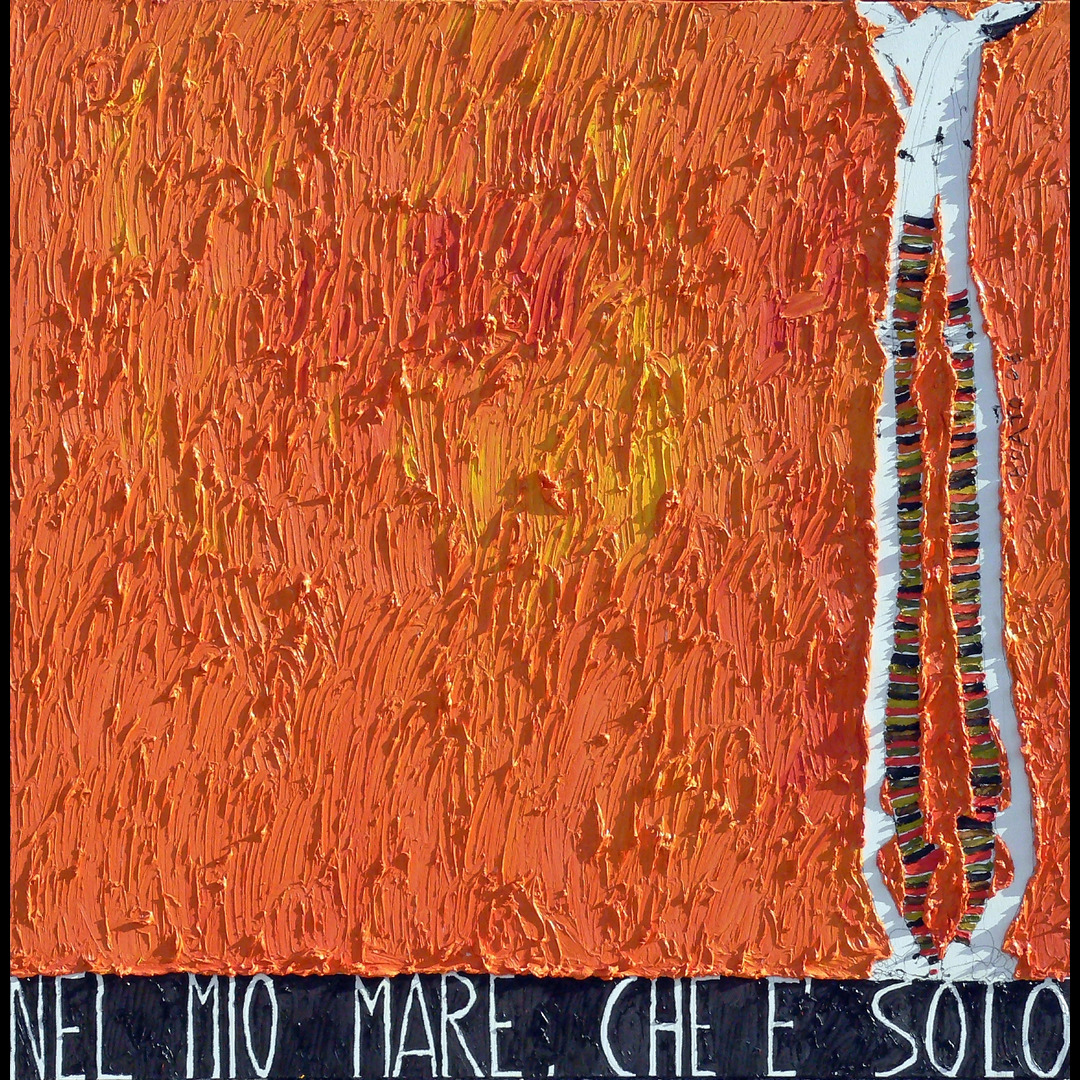

Supplement: Supplementary file 2 [file Presentation2.ZIP › 13 Insogno, 2006, olio su tela, 100x100-A-1080.jpg]

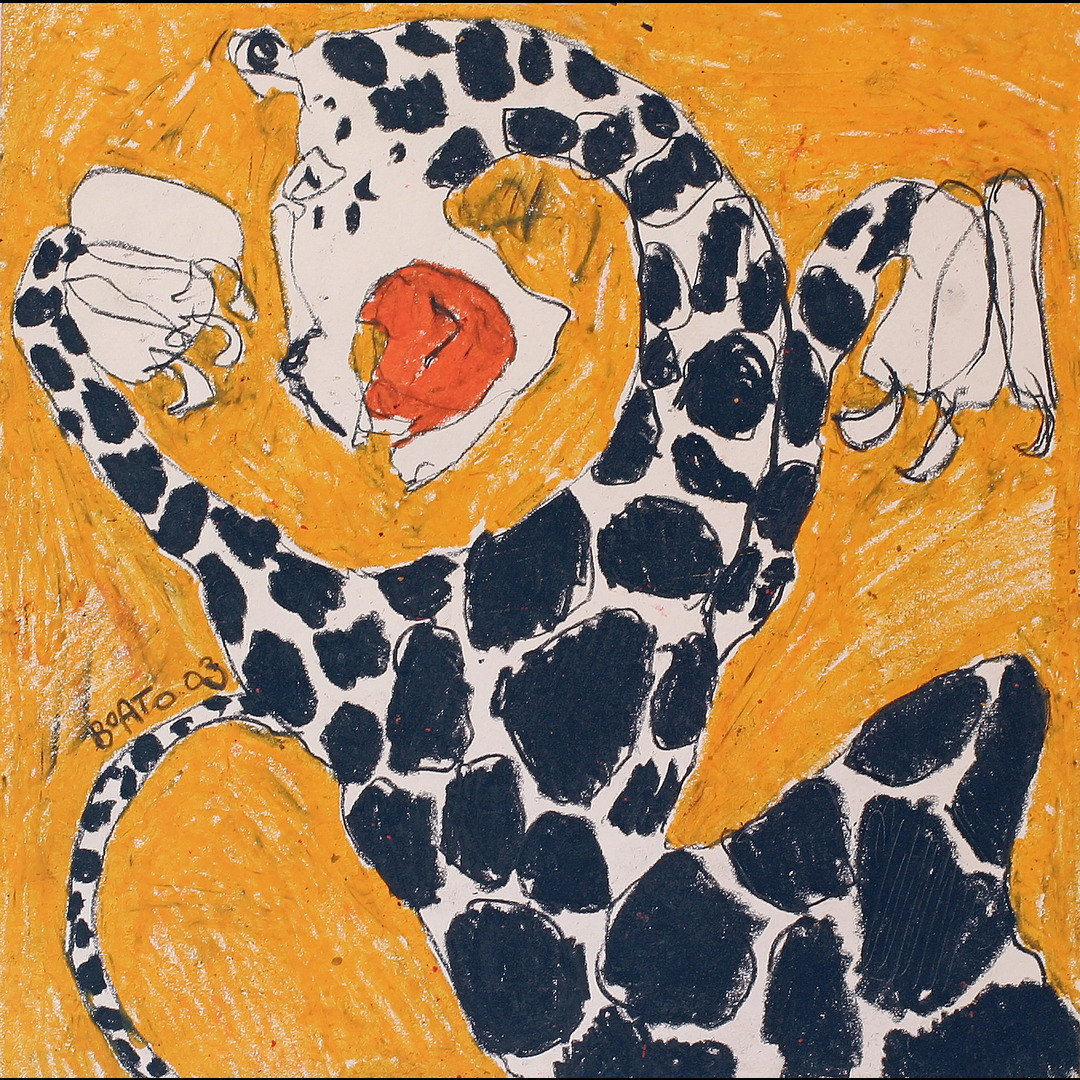

Supplement: Supplementary file 2 [file Presentation2.ZIP › 14 Leopardo, Matteo Boato, pastello ad olio su carta, 2003-A-1080.jpg]

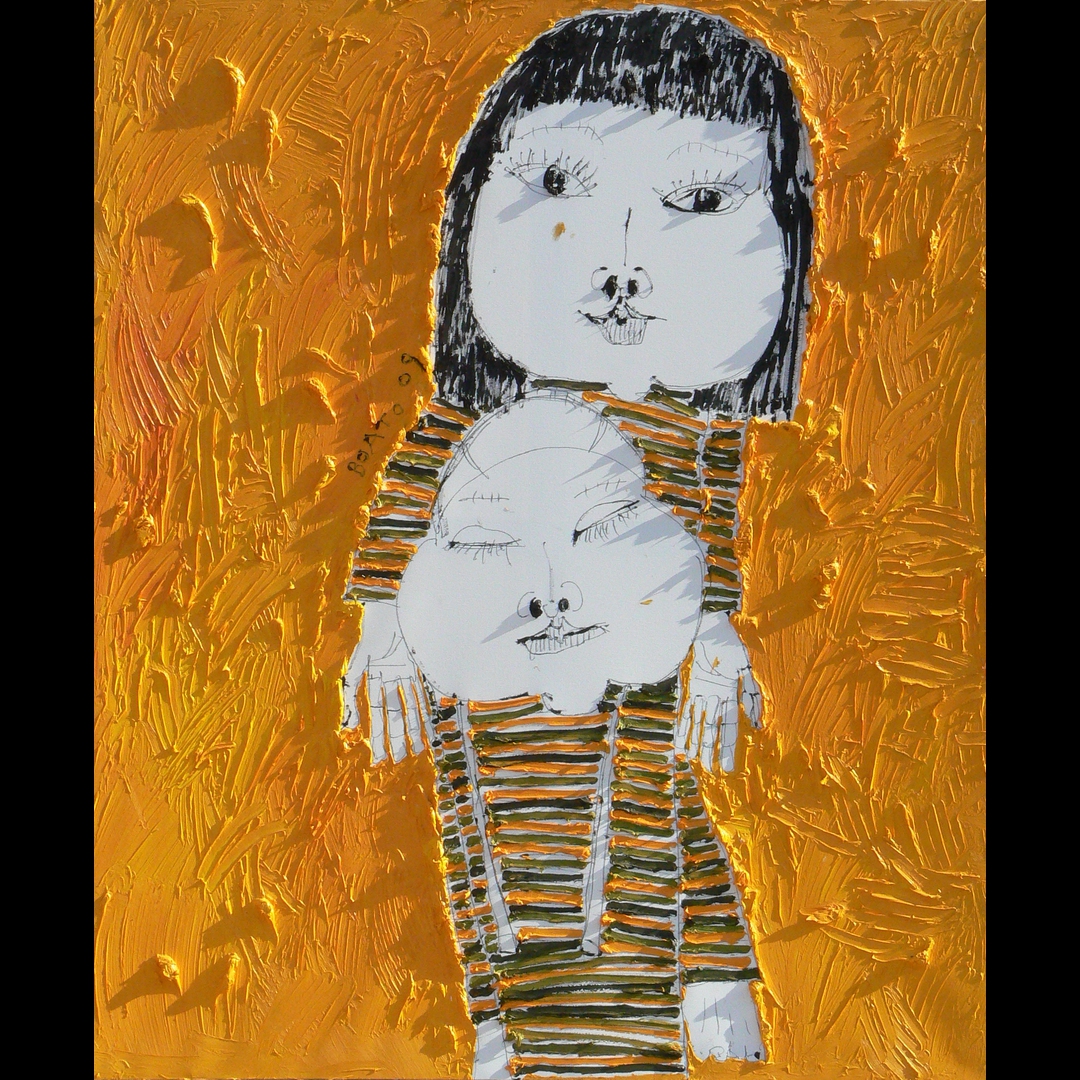

Supplement: Supplementary file 2 [file Presentation2.ZIP › 15 Matilada e Beatrice,2009,olio su tela, 100x120-1080.jpg]
